# Zeolites in Adsorption Processes: State of the Art and Future Prospects

**DOI:** 10.1021/acs.chemrev.2c00140

**Published:** 2022-10-19

**Authors:** Eduardo Pérez-Botella, Susana Valencia, Fernando Rey

**Affiliations:** Instituto de Tecnología Química (ITQ), Universitat Politècnica de València (UPV) - Consejo Superior de Investigaciones Científicas (CSIC), Av. de los Naranjos s/n, 46022 Valencia, Spain

## Abstract

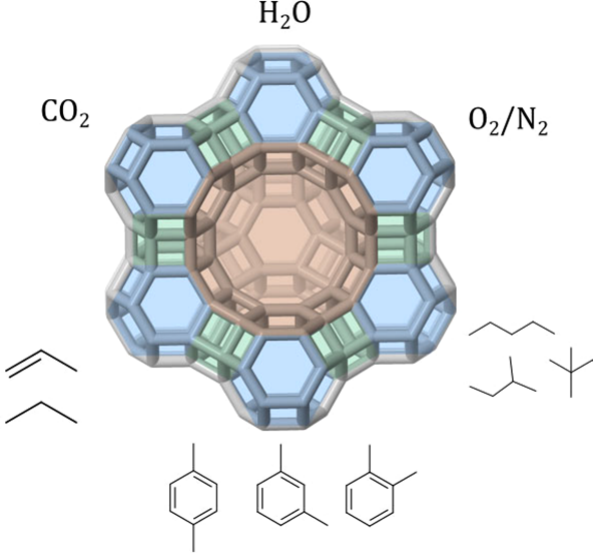

Zeolites have been widely used as catalysts, ion exchangers, and adsorbents since their industrial breakthrough in the 1950s and continue to be state-of the-art adsorbents in many separation processes. Furthermore, their properties make them materials of choice for developing and emerging separation applications. The aim of this review is to put into context the relevance of zeolites and their use and prospects in adsorption technology. It has been divided into three different sections, i.e., zeolites, adsorption on nanoporous materials, and chemical separations by zeolites. In the first section, zeolites are explained in terms of their structure, composition, preparation, and properties, and a brief review of their applications is given. In the second section, the fundamentals of adsorption science are presented, with special attention to its industrial application and our case of interest, which is adsorption on zeolites. Finally, the state-of-the-art relevant separations related to chemical and energy production, in which zeolites have a practical or potential applicability, are presented. The replacement of some of the current separation methods by optimized adsorption processes using zeolites could mean an improvement in terms of sustainability and energy savings. Different separation mechanisms and the underlying adsorption properties that make zeolites interesting for these applications are discussed.

## 1 Introduction

Separation processes are of extremely high importance for our society, comprising 10–15% of the global energy use and 40–90% of the total process cost.^1,2^ On one hand, most of the existing chemical processes do not yield products with the desired purity, directly suitable for further use. This is the case of petrochemical products or alcohols produced by fermentation, both of which require purification steps after being produced.^2,3^ On the other hand, the separation and purification of naturally occurring mixtures, such as air or natural gas, is of high environmental, economical, and/or practical interest.^4−6^

Until a not too distant past, many separation processes did not take the ecological axis into account, mainly due to the wide availability of fossil fuels as an energy source and the lack of environmental awareness.^1^ As climate change and resource scarcity threaten to become more severe, cost reduction gradually aligns with sustainability objectives, and the focus of the industry is drawn from traditional separation procedures, with distillation being the main exponent, to alternative technologies or process designs. The development of improved methods for separations, such as hydrocarbon separations, the separation of olefins from paraffins, or the removal of carbon dioxide from industrial exhaust gas or from the atmosphere, could have an enormous impact on our progress toward sustainability.^1^

Adsorption represents one of the possible alternative separation technologies for many fluid mixtures. It is a mature technology that has been used for separation applications, such as water treatment, drying, hydrogen purification, air separation, or hydrocarbon separation.^7^ At the same time, the versatility of the technique, i.e., the growing range of conditions and process designs available, and, most of all, the vast dimension of possible adsorbents make it an ever evolving technology that is gaining the interest and recognition of the academic and industrial communities.

The industrial development of adsorption processes is tightly bound to the commercialization of the first zeolitic materials.^8−10^ Zeolites are microporous crystalline aluminosilicates with well-defined pore sizes, and for this reason, they are able to discriminate between molecules of high practical interest, such as CO_2_, ethylene, isobutene, or xylenes, with a precision of tenths of Å (10^–11^ m).^11^ Additionally, their structural and compositional richness allows for fine-tuning of their adsorption properties to address a targeted separation. They have been successfully applied to drying of gases and liquids,^12^ to the separation of nitrogen from oxygen,^4^ to the separation of linear from branched hydrocarbons,^13^ and to the separation of xylene isomers among other separations.^14^ Nonetheless, they are potentially applicable to many different separations which are currently performed by different technologies, such as CO_2_ removal, olefin–paraffin separation, and the separation of hydrogen isotopes, or which still are in development, e.g., the separation of mono- from multibranched hydrocarbons.

In this review, we present the state-of-the-art zeolites as adsorbents and their potential to address separations of current industrial interest. Section 2 serves as an introduction to this interesting type of materials and puts special attention on the relationship between their synthesis and preparation, their physical and chemical properties, and their applications in ion exchange, catalysis, and adsorption. Section 3 provides basic knowledge that a reader new to the field of adsorption will find useful to understand the concepts presented later on and their implications. Finally, Section 4 presents a selection of relevant separations, ordered by complexity of the targeted molecules, for which zeolites are already in use or potentially applicable adsorbents. The advantages and limitations of these materials are critically reviewed for applications under development, and promising results and trends are highlighted.

We aim this review at a broad audience interested in chemical separations. Readers with experience in zeolites and catalysis will be able to get into the field of zeolites as adsorbents and to find out about promising applications which may be accessible to them. On the other hand, readers with knowledge in adsorption may get an overview on the variety of relevant separations currently being targeted and also on the vast possibilities that zeolitic adsorbents offer. Readers with interest in pursuing a specific separation out of the ones included here will be provided with a solid starting point and a rich and updated literature background.

## 2 Zeolites

Zeolites are crystalline microporous aluminosilicates widely used as catalysts, adsorbents, and ion exchangers. They belong to the tectosilicate-type minerals, and some of them occur naturally. Their well-defined pore size, compositional tunability, thermal stability, and commercial availability since the 1950s^15−18^ have boosted their use in industrial and domestic applications, which take advantage of their unique properties. In the following sections a short historic review of zeolites and their use (Section 2.1) is first presented, then their structure and composition (Section 2.2), from which their properties derive, are explained. Later, the general synthetic procedure to obtain these materials (Section 2.3) is summarized, and finally their properties and most important applications are briefly reviewed (Section 2.4).

### 2.1 A Short History of Zeolites

The term zeolite was coined by the Swedish mineralogist Axel F. Cronstedt in 1756^19^ after he observed froth forming on the surface of a mineral sample upon heating.^20^ The mineral was apparently “boiling”, and thus, he named it “zeolite”, from the Greek *zein* “to boil” and *lithos* “stone”. Later, this phenomenon was ascribed to the presence of hydration water inside of the pores of the mineral, which is liberated upon heating. Cronstedt’s mineral has been identified as a mixture of stellerite and stilbite.^20^

For the next years no noticeable discovery was made by chemists in reference to zeolites, and it was not until 1840 that Damour demonstrated the reversible hydration and dehydration of these materials.^21^ The first demonstration of the cation-exchange properties of natural zeolites (chabazite and natrolite) was in 1858,^22^ and the first report on zeolite synthesis was in 1862, with the synthesis of levyne.^23^ The first industrial success of these materials was based on their ion-exchange properties, as water softeners for laundry compositions.^24−26^ This still remains one of their major applications.

The adsorption of species other than water was first reported by Friedel in 1896^27^ and further studied by Grandjean in 1909.^28^ Selective adsorption and exclusion of molecules, i.e., the molecular sieve effect, was first described in 1924 by Weigel and Steinhoff, who observed how water, methanol, ethanol, and formic acid were adsorbed on chabazite, while acetone, diethyl ether, and benzene were excluded.^29^ This effect could not be explained until the structural porosity of zeolites was described following the first structural elucidations of these materials.^30−32^ In 1932, McBain coined the term “molecular sieve”, referring to zeolites and their very high selectivity when applied to adsorption processes.^33^ McBain’s work was a turning point in zeolite science, as it encouraged a young researcher, Richard M. Barrer, currently considered the father of zeolite science, to dive into these materials’ field of research.^34^

Barrer studied the separation of mixtures of many different molecules on zeolites and realized the great potential of these materials as adsorbents for separation processes. Over the next 20 years he successfully attempted the synthesis of zeolites by mimicking the crystallization conditions of natural zeolites (hydrothermal, i.e., alkaline media and temperature above 200 °C), thus obtaining some synthetic analogues of natural zeolites, such as chabazite and mordenite,^35−37^ and others with no natural counterpart, which he named as zeolites P and Q.^38−40^

In 1949 he described the alkaline–ammonium cation exchange in zeolites followed by calcination as a strategy to obtain them in their proton-exchanged form.^41^ During his time at the Imperial College (1954–1976), Prof. R. M. Barrer achieved the first zeolite synthesis using tetraalkylammonium cations,^42−44^ which, in retrospect, has turned out to be the most fruitful strategy for obtaining new zeolitic materials to the present time and still remains state of the art (see Section 2.3).^45−48^

**Figure 1:**
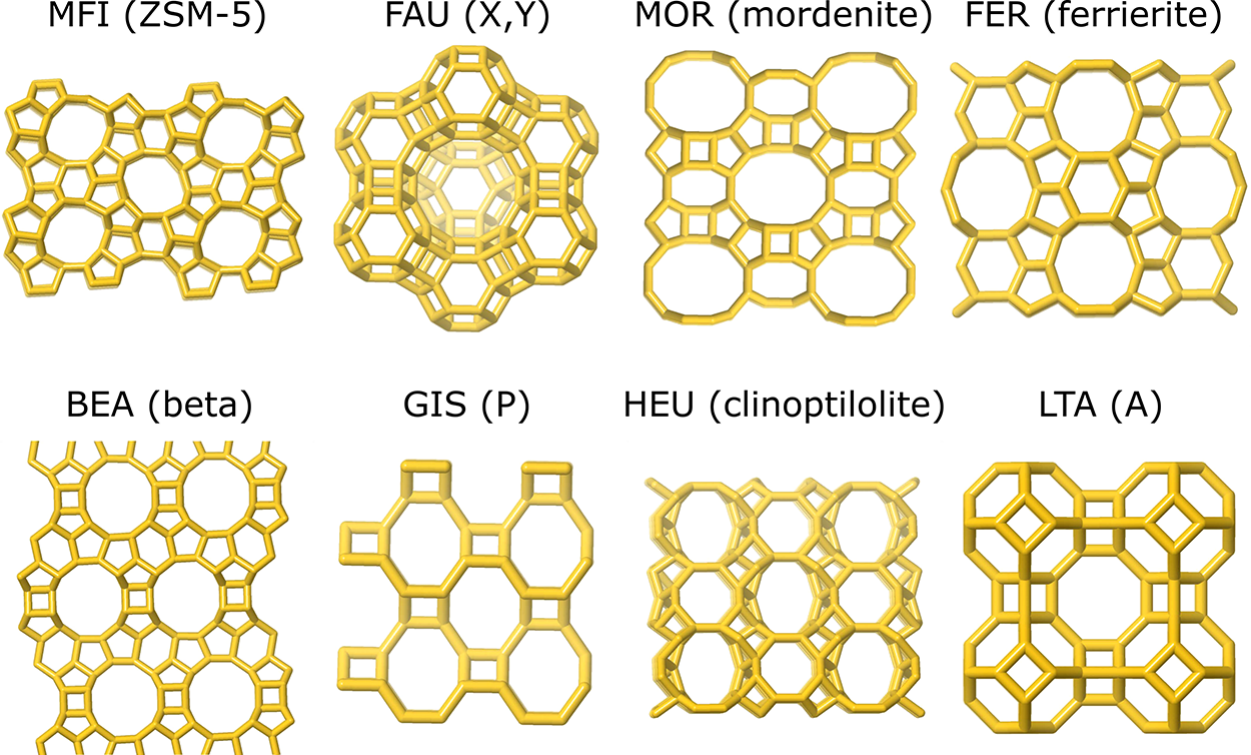
Front view of the main pores of selected zeolites. The three letter codes written above each picture are the official names given to each structure by the International Zeolite Association. Next to the name, the common name of the zeolite is indicated in parentheses.

Barrer’s discoveries attracted the interest of the industry on zeolites and resulted in the development of commercial zeolite production and their application. The most relevant of these contributions were by Union Carbide in the USA within a research program which started in 1949, with Barrer as an academic consultant. Robert M. Milton enrolled in this research program with the objective to develop an adsorption method to separate N_2_ from O_2_ instead of traditional cryogenic distillation. Inspired by the works on molecular sieving by McBain and Barrer, he attempted this separation using chabazite as the adsorbent. While trying to obtain this zeolite (which he managed by 1950^49^), he varied the synthesis conditions by means of lowering the temperature to 25–150 °C and using more reactive silica sources and more alkaline media. This led to the rapid obtention of zeolites A and X (see Figure 1), along with 14 other new zeolite materials.^15,34^ Donald W. Breck joined Milton’s group in 1951, and he discovered zeolite Y in 1954. This zeolite is isostructural to zeolite X but presents lower Al content. In the following years, the zeolite research group at Union Carbide developed the scaling up of these syntheses, and in 1954 zeolites A and X were commercialized for adsorption applications.^15,17,18,49^ Meanwhile, 24 new zeolitic materials were discovered by this group.^15,50−52^ A simplified structural depiction of some of the most industrially important zeolites, including catalysis’ “big 5”, i.e., ZSM-5, X, Y, mordenite, ferrierite, and beta, is presented in Figure 1.

The breakthrough in zeolite science in the 1950s mostly led by Union Carbide boosted other companies’ interest in these materials, as well. In the next years, many new and modified zeolites were discovered and used in adsorption, ion-exchange, and catalysis applications. More information on the use of zeolites will be given in Section 2.4.

### 2.2 Structure and Composition

**Figure 2:**
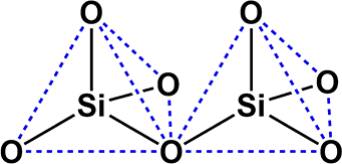
Two corner-sharing SiO_4_ tetrahedra.

The properties that make zeolites especially useful as catalysts and adsorbents stem from their crystalline structure and also from their composition. Zeolites are crystalline microporous aluminosilicates, the framework of which consists of corner-sharing TO_4_ tetrahedra (see Figure 2), where T are tipically Si or Al atoms. The empirical formula of an aluminosilicate zeolite can be represented by , where *x* = *yz* and is most frequently limited to 0 ≤ *x* ≤ 0.5, a phenomenon known as Löwenstein’s rule.^53^ The presence of tetrahedrally coordinated Al atoms leads to negative charges in the framework that are compensated by extraframework cationic species, represented in the formula above as M^*z*+^.^54^ These cations are located inside the pores and cavities of the zeolite framework and can be of an organic (typically alkylammonium) or inorganic nature (alkaline, alkaline earth, and other metals), depending on the synthesis conditions and on whether the material has been subjected to postsynthesis treatments (calcination, ion exchange). Natural zeolites and many synthetic zeolites contain metallic cations, which are usually hydrated and account for Cronstedt’s discovery.

According to their Si/Al ratio, aluminosilicate zeolites can be classified as low-silica zeolites (Si/Al ratios below 2, highly polar), medium-silica zeolites (Si/Al ratio between 2 and 5, intermediate polarity), and high-silica zeolites (Si/Al > 5).^55^ There is as well the special case of zeolites with no aluminum at all, known as zeosils or pure-silica zeolites. Logically, they contain no extraframework cations. Pure-silica and high-silica zeolites are under intense research, as they present a hydrophobic surface and generally also larger thermal and chemical stabilities than traditional zeolites, which make them very appealing for adsorption applications.^16,56−58^

On the other hand, the T atoms can be different from Si and Al. There are many compositional variants of zeolites which present structures analogous to or different from aluminosilicate zeolites. An advanced “chemistry search” in the Database of Zeolite Structures^59^ is a straightforward way to obtain a quick overview on the rich compositional variability of zeolites. Apart from Si and Al, which are not necessarily present in all zeolite-like materials (zeotypes), other atoms can be found in tetrahedral coordination in the framework, such as B, Be, Co, Fe, Ga, Ge, Mg, P, Ti, and Zn. It must be noted that the presence of some of these “heteroatoms” can facilitate the crystallization of specific structures which are otherwise not achievable and also of materials with different chemical properties.

For instance, isomorphic B incorporation in zeolites has been frequently achieved, resulting in acid microporous catalysts of milder acid strength than Al-containing zeolites.^60,61^ Importantly, very often, B-silicate zeolites are produced with different structures than Al-substituted zeolites.^62−65^ Also, the relatively high lability of B in framework positions allows its isomorphic exchange by Al through secondary synthesis treatments, enabling Al incorporation in zeolites that can not be synthesized directly as Al silicates.^66^

Another interesting case of the structure-directing effect of framework heteroatoms is that of Ge, which can replace Si. It has been observed that Ge incorporation very often directs to the formation of double four ring (D4r) containing zeolites, where Ge tends to be placed.^67−71^ The preferential location and strong directing effect toward D4r-containing zeolites have been attributed to the larger germanium atomic radius,^72−74^ which introduces framework flexibility and high tolerance in the crystal structure to relatively acute T–O–T bond angles resulting in zeolites having low framework densities as predicted by Brunner.^75^

**Figure 3:**
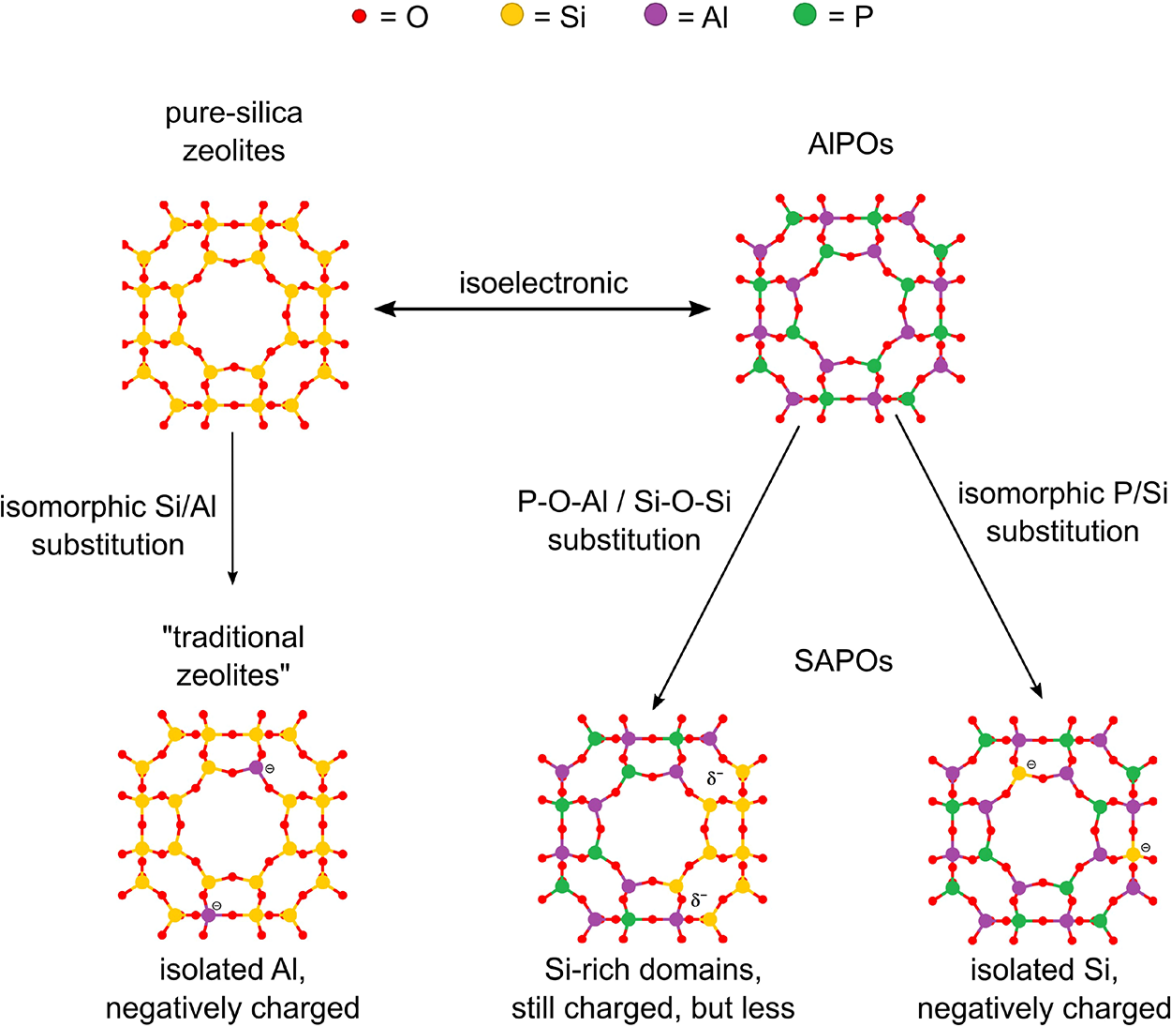
Isomorphic substitution scheme of some compositional variants of zeolites, exemplified in an LTA cavity.

Aluminophosphate (AlPO) materials are isoelectronic with pure-silica zeolites and present a perfectly alternating sequence of AlO_4_ and PO_4_ tetrahedra. They have proven interesting for adsorption and heat exchange applications, even though frequently they present more limited thermal and chemical stabilities if compared to other zeolites.^68,76^ There is a series of AlPO-related materials, which are in concept heteroatom-substituted AlPOs.^77,78^ The possible “heteroatoms” include Si, Fe, Mg, Mn, Co, Zn, Ti, V, and/or Cr among others.^79^ In silicoaluminophosphate (SAPO) materials, part of the T positions of the framework are occupied by Si atoms. Silicon substitution in SAPOs follows conceptually more complicated patterns than Al substitution in aluminosilicate zeolites, as Si can “replace” a single P atom (isolated Si), but also larger framework fragments, yielding what is known as Si islands or Si-rich domains (see Figure 3). SAPOs have found use in adsorption and catalysis. Metal aluminophosphate and metal silicoaluminophosphate materials have been widely studied as catalysts.^78,80−83^

Depending on the T atoms present in a framework, the chemical and physical properties of the material will vary. The presence of atoms with redox properties, such as Ti, Co, Fe, or V, may have a great influence on the redox chemistry of the material.^79,84^ The acidity/basicity of specific adsorption sites depends as well on the composition of the framework. The ratio of tetravalent (Si, Ge, etc.) to trivalent (Al, B, etc.) atoms, usually the Si/Al ratio, largely defines the polarity of the material. For instance, zeolites with a higher Al content (also known as low-silica zeolites) adsorb larger amounts of polar compounds, such as water, than high- or pure-silica zeolites.^55^ Additionally, the charge-balancing extraframework cations can contribute with their specific chemical properties to the chemistry of the material.^85^

However, the most important feature of zeolites and the one that has made them interesting for any application, since they were first studied in detail by Barrer, is their structure-derived porosity. The flexibility of the T–O–T angle allows for different spatial dispositions of the tetrahedra,^45^ thus resulting in a large number (millions) of different hypothetical porous structures.^86^ More than 250 different zeolitic structures are known to exist at the present time, of which some can be found in nature and others are synthetic. Each structure is given a three-letter code and registered in the Database of Zeolite Structures,^59^ where a thorough structural and crystallographic description is provided.

**Figure 4:**
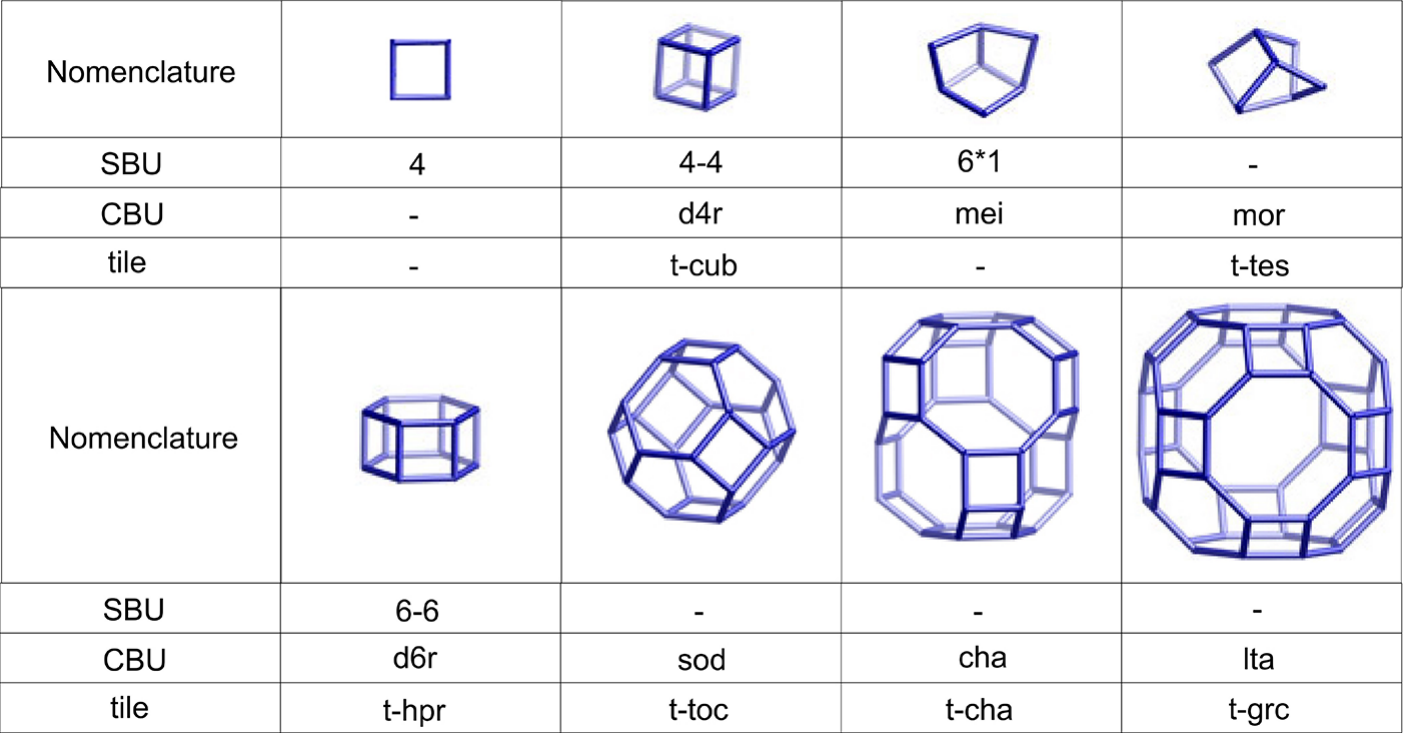
Examples of building units and their possible names, according to the IZA Structure Commission.^59^ Vertices represent T atoms. Oxygen atoms are not depicted.

The structural description of zeolites is usually performed in terms of their building units. The TO_4_ tetrahedra, i.e., the primary building units of zeolites, can be linked following different arrangements, which result in secondary building units (SBUs), composite building units (CBUs), or the so-called “tiles”. SBUs contain a maximum number of 16 T atoms and were initially intended to be the sole descriptor of zeolite structures; i.e., a single SBU type (of which a total number of 23 are listed in the Database of Zeolite Structures^59^) should suffice for the description of each framework. At the same time, different SBUs could be used to describe a single framework, and different frameworks could be described using the same SBU. However, in 2007 it was realized that the SBUs were insufficient for the universal description of zeolite structures, and the listing of new SBUs ceased. Instead, the broader concepts of CBU and/or tiles were introduced and recommended. It must be noted that there is an overlap between these descriptors, and some arrangements of tetrahedra can belong to two or all three of these kinds of descriptors. For instance, the double 4-ring belongs to all three of them and is named differently in each case (“4–4” according to the SBU nomenclature, “d4r” according to the CBU nomenclature, and “t-cub” according to the tile nomenclature). Examples of typical building units are given in Figure 4.

A more general notation of the CBUs, also applicable for new structures and building units, follows the scheme , where *m* is the number of *n*-rings defining the polyhedron and *∑m*_*i*_ the total number of faces. Thus, the *d6r* building unit could be expressed more generally as [4^6^6^2^] and the *sod* building unit as [4^6^6^8^].^87^ In some cases, instead of polyhedral building units, chain building units may be useful for structural description.

Another approach for the structural description of zeolites is based on the size, connectivity, topology, and geometry of their pore systems. The pores are the void spaces inside the framework that are not occupied by framework atoms. These pores can be accessible or inaccessible to molecules of various sizes, depending on how they are connected and the window size (the *n*-rings are called windows). Polyhedral units with windows smaller than or equal to 6R are named cages, and only a few very small molecules, e.g., water, can penetrate these. The *sod* building unit shown in Figure 4 is an example of a cage and receives the name of a sodalite cage or β-cage. Finite polyhedra with at least one of its faces consisting of a window larger than 6R are called cavities, an example of which is the *lta* building unit shown in Figure 4, also called the α-cavity. Pores that extend indefinitely in one direction and whose size allows for diffusion of guest molecules along its length are called channels. Zeolites with pore systems which present channels in only one direction or nonintersecting channels in different directions are called unidirectional. When channels in different directions intersect, they can form bidirectional or tridirectional channel systems.

According to the minimum window size of the largest pores present in their structure, zeolites can be classified as follows:^54,55,87,88^

- Small pore zeolites have a minimum pore diameter between 3 and 5 Å, which corresponds to rings consisting of 8–9 TO_4_ tetrahedra (8–9R).
- Medium pore zeolites have a minimum pore diameter between 5 and 6 Å, which corresponds to 10-rings (10R).
- Large pore zeolites have a minimum pore diameter between 6 and 7.5 Å, which corresponds to 12R.
- Extra large pore zeolites have a minimum pore diameter above 7.5 Å, which corresponds to rings of more than 12 tetrahedra.

Within these groups, there are many structures with different pore sizes and shapes. If a zeolite presents more than one kind of pore, it will be classified according to the largest pore present. For example, the STW framework presents intersecting channels with different minimum window sizes, i.e., 8R and 10R, and is considered a medium pore zeolite. The topology of the pore system can be of high importance as well, as it has a large impact on the interaction and diffusion of molecules inside the pores.

### 2.3 Preparation of Zeolites

#### 2.3.1 Hydrothermal Synthesis of Zeolites

The synthesis of zeolites is usually performed following the hydrothermal method, which mimics the natural conditions that lead to the crystallization of zeolites. This includes a source of the T atoms (in nature, it is volcanic ash and volcanoclastic materials), a structure-directing agent (SDA; in nature, usually alkaline or alkaline-earth cations), a mineralizing agent (usually alkaline aqueous solutions), temperatures below 600 °C, and autogenous pressures.^89,90^ Through imitation of the natural process, some zeolites were obtained, mostly analogues of minerals existing in nature. However, it was by modifying it that the structural and compositional richness of these materials started to become apparent.

The synthetic processes that have led to the most discoveries of new zeolitic structures and compositional variants follow these guidelines in general terms but present many singularities. The T atom source is usually an oxidized form of the T atom. For instance, typical Si sources are amorphous, fumed, or colloidal silica, silicates, alkyl silicates, and other zeolites/materials. These kinds of Si sources with enhanced surface area and solubility were a key for success when Milton and co-workers^49^ started the search for new zeolites in 1949. Typical Al sources include different kinds of alumina, aluminum hydroxides, aluminum alkoxides, and aluminates. In the case of AlPOs and SAPOs, P is most frequently added as phosphoric acid.^91^

The role of the SDAs is of large importance, as they not only promote the crystallization of specific structures but also may remain inside the pores of the final material to some extent and act as charge-balancing ions. The first SDAs that were used in zeolite syntheses were cations of inorganic nature, such as Na^+^, Ca^2+^, or K^+^. Nonetheless, the most remarkable type of SDAs and the ones that meant a breakthrough in zeolite science are organic SDAs (OSDAs), which are in most cases amines and alkylammonium cations.^45−48^ These OSDAs were initially referred to as “templates”,^92,93^ a term which is still frequently (and inaccurately) used to address OSDAs in general. It has its origin in the so-called “template effect” that some OSDAs possess, in which their presence in the synthesis gel leads to the crystallization of a specific structure with matching topological features.^92^ Other molecule types, such as alkylphosphonium cations, alkylsulfonium cations, phosphazenes, crown macrocycles, metal complexes, and self-assembled molecules, have been used as OSDAs but with a quantitatively more modest degree of success than nitrogen OSDAs.^94^ The way in which these OSDAs favor the crystallization of a specific structure is not yet fully understood, despite the large research effort put into it.^46,47,95,96^ However, the rational design of OSDAs in the search for particular zeolites has given good results in some cases.^57,97−100^ In this sense, recent advances in data mining and artificial intelligence have allowed us to computationally predict to some extent which OSDAs may lead to the crystallization of targeted zeolite structures.^97,99,101^ In general terms, linear OSDAs favor the crystallization of 1D structures; branched OSDAs favor the crystallization of interconnected 2D and 3D structures; and bulky OSDAs favor the crystallization of structures possessing cavities. The lower charge density of the OSDAs in comparison with the alkaline and alkaline-earth cations allows for less charged frameworks, thus facilitating the obtention of final materials with a higher Si/Al ratio.^47,55^ More than one kind of inorganic or organic SDA may be present in the synthesis gel, and both may act as SDAs; however, they also may have been added to increase basicity, as explained below. Additionally, the T atoms present in the synthesis gel can have a structure-directing effect, too, as they may favor the crystallization of structures bearing specific CBUs. This is the case for Ge, or Be, which favors D4R and 3R, respectively.^48^

On the other hand, there is an increasing interest in reducing the amount of OSDA needed for the synthesis of certain zeolites (especially high silica) or even to find ways to recycle it or dispense with it.^102^ By doing so, the environmental impact of zeolite production as well as its cost would be lower, thus easing the requirements for scale-up.^48,103^ The OSDA-free synthesis of zeolites was reported for the first time in 1985 for high-silica ZSM-5,^104^ thus being a rare example of such syntheses until the publication of Xie et al. in 2008 reporting the crystallization of zeolite Beta in the absence of OSDA.^105^ In this report, the use of seeds (see below) of zeolite Beta was crucial for growing the desired zeolite. Since then, a large number of researchers have devoted large efforts to expanding the range of zeolites synthesized through OSDA-free synthesis routes^106−108^ and also to understanding the synthesis parameters that govern the growth of zeolites in such conditions.^109−111^

The mineralizing agent intervenes directly in the breaking and formation of T–O–T bonds and helps to establish a dynamic equilibrium that ends in the formation of the zeolite.^96,112^ Possible mineralizing agents are the hydroxide and fluoride anions.^113^ Hydroxide anions are the most widely used mineralizing agent, and they are frequently added along with the SDA. If an extra amount of hydroxide anions is needed, it is usual that inorganic (NaOH, KOH, NH_4_OH) bases are used for low-silica zeolites and organic (amines, alkylammonium) bases for high- and pure-silica zeolites. The source of fluoride anions can be hydrofluoric acid, which in turn decreases the pH of the gel (this may be desirable for preventing OSDA decomposition), or ammonium fluoride. Some zeolites have been synthesized both from gels containing hydroxide and from fluoride, and there are interesting consequences to the use of one or the other. The fluoride anion has in some way a structure-directing effect, too, in which it favors the formation of certain CBUs and phases with lower densities.^114,115^ On the other hand, zeolites synthesized from fluoride-containing gels tend to present an extremely low amount of defects.^116^ The H_2_O/SiO_2_ ratio is important as well, especially in high silica gels in fluoride media, as it affects which kind of frameworks will be obtainable based on the density of the final material and the size of the crystals.^113,117,118^

Another way of influencing the synthesis outcome is to introduce crystal seeds of a certain zeolite “parent” structure in the synthesis gel. The seeds can be preserved or dissolved into anionic species with one or more T atoms (sacrificial seeding).^119^ When preserved, the seeds promote the crystallization of their same structure, as the nucleation step is skipped and the crystals can start to grow immediately.^96^ When sacrificed, the seeds promote the crystallization of a phase that may or may not share structural resemblance with the parent structure.

Crystallization temperature and time have a decisive effect in the synthesis of zeolites.^49,91,96,112^ Higher temperatures and longer crystallization times favor the obtention of more dense, usually more stable phases instead of more open phases. On the contrary, the pressure in the gas phase does not seem to have any effect on the synthesis.^49^ Other synthesis parameters that have a remarkable influence on the product obtained are aging of the gel at lower temperature prior to the hydrothermal process and stirring/rotation speed during the crystallization.^96,112^

As can be seen, there are many different variables that affect the results of the hydrothermal synthesis of zeolites. The complexity of these heterogeneous systems has not allowed for a full rationalization of the crystallization mechanisms or of the specific conditions that lead to the crystallization of a specific phase with a well-defined crystal size and composition. However, general trends on how each and every one of these parameters affect the synthesis outcome are understood and applied to new synthetic processes in order to reduce the range of possible results.

#### 2.3.2 Novel Methods and Trends in Zeolite Synthesis

The advances in zeolite synthesis are a major drive for their application in new and established processes. Whereas the previous section deals with the conventional synthesis methods, in this section, an overview on more recent synthetic trends and methods will be given, including nonconventional synthesis methods. The production of hierarchical zeolites and nanocrystalline zeolites and the control of heteroatom distribution will also be discussed here.

Modification of parameters within the context of hydrothermal synthesis has been a relatively successful strategy to obtain new zeolitic materials throughout the years. However, the rate of discovery of new zeolitic structures and materials can be accelerated through the implementation of nonconventional techniques, such as interzeolite and topotactic conversions, ionothermal synthesis, and microwave- or radical-assisted synthesis.^102,120^ Zeolite interconversion takes place in gels, where the silica source is replaced by a parent zeolite which shares common building units with the target zeolite, an example of which is the transformation of faujasite into chabazite.^121−123^ On the other hand, new zeolites can be obtained by topotactic transformation in which some labile atoms are removed by mild secondary treatments followed by condensation or pillarization of the remaining zeolitic layers, providing new fully ordered microporous materials.^72,103,124−126^ Solid-phase transformation by applying high pressure on the zeolite has been also described to transform the structure of one parent zeolite into another zeolite of different topology.^127,128^ The presence of radicals in the synthesis media has been claimed to accelerate the crystallization of zeolites^129^ as has been shown for microwave heating,^102,130^ while using ionic liquids as the solvent instead of water, i.e., ionothermal synthesis, decreases the operating pressure, allowing the synthesis to be carried out at ambient pressure.^131−133^

Diffusion of molecules inside the pores of a zeolite is of paramount importance in adsorption processes (see Section 3.1.2) since the final productivity of the adsorption/separation unit depends on the adsorption rate of the molecule across the adsorbent. The rate at which a molecule gets adsorbed or moves through the pores of a zeolite can be controlled by modifying its diffusion path, which in practice can be done by changing the crystal size or the pore size. The reduction of average crystal size of a zeolite is mostly done directly by hydrothermal synthesis promoting nucleation versus crystal growth,^99,134−138^ although other nonconventional methods have been applied as well.^139^ The aggregation of these nanosized crystals gives rise to “hierarchical” zeolites with an effective meso- or even macroporosity.^140^ Moreover, changes in the effective pore size can directly be induced by ion exchange, functionalization, or steaming, all of which are explained in Section 2.3.3. Steaming can lead to the dealumination and/or desilication of the zeolitic material, i.e., the partial dissolution of the framework.^141^ Accordingly, these methods are used to produce hierarchical zeolites, which feature not only their original microporosity but also additional mesoporosity. The presence of a multimodal pore size distribution of micro-, meso-, and/or macropores leads to an increased accessible surface area, shorter diffusion distances, and higher mass transfer rate when used as adsorbents in separation/adsorption processes. Therefore, hierarchical zeolites have been extensively studied in the past decade.^140,142−145^

Heteroatom distribution in zeolitic materials has a large influence on their interaction with molecules. It has been found that selectivity in many acid-catalyzed reactions by Al-containing zeolites strongly depends on the Al distribution in the zeolite framework, which can be controlled by modifying the synthesis conditions.^99,138,146−149^ The charge density, size, and concentration of the SDA play an important role in this sense.^138,146,150^ The distribution of Al has been shown to have an effect on the adsorption properties of zeolites, as well. For instance, when the aluminum is evenly distributed throughout the framework, the adsorption capacity of aluminosilicate zeolites is enhanced and the adsorption heat remains constant.^150,151^ In the case of SAPOs, the Si distribution can be controlled by modifying the synthesis conditions and also has an important effect on the catalytic and adsorptive behavior of the materials.^81,82,152^

#### 2.3.3 Postsynthetic Modification of Zeolites

Even though many zeolites can be obtained by direct synthesis with tailored composition and structure, it is common that further processing, i.e., postsynthetic treatment, is needed to achieve the desired properties in the final material. Ion exchange, desilication, surface functionalization, calcination, and steaming are some of the most frequently used methods.^143,153,154^

Ion exchange of aluminosilicate materials allows us to modify their acid–base, redox, and textural properties (pore sizes and interaction with adsorbates). It is usually performed in an aqueous solution with a high concentration of the cationic species to be exchanged. After reaching equilibrium, the zeolite is filtered, washed, and dried and can be subject to further exchange or modifications. Exchange of small cations, such as metals or ammonium, is the usual case. If the zeolite pores are too narrow for the extraframework species to diffuse, ion exchange may not be possible, which is usually the case for OSDAs.

Functionalization of zeolite surfaces allows us to introduce new chemical and/or physical properties to zeolitic materials. Functionalization by supporting transition and noble metals on zeolitic materials has been widely used for catalyst preparation (see Section 2.4.2), as it allows us to obtain high surface area and highly chemically active catalysts. In this case, the reactivity of the metallic species is (partially) conveyed to the final material. In the case of adsorbents, Ag and Cu have been the most frequently supported species, especially for their use in olefin, hydrogen, and carbon monoxide adsorption.^155,156^ Supported metal zeolitic materials are most frequently produced by impregnation or ion exchange and, more recently, by chemical vapor deposition.^153,154^

Another type of functionalization involves grafting of nonmetallic functional groups to the zeolite surface, examples of which include inorganic acids, amines, and silanes. Amine grafing on zeolites has gained attention in the last years for carbon capture applications, although in this case the protagonists are still amine-grafted mesoporous silicas.^157,158^ The amine groups help decrease the hydrophilicity of the zeolite and keeps good affinity toward CO_2_, thus improving the recyclability of the material.

Silanization is a type of surface functionalization of zeolites that can be used to selectively deactivate external surface acidity and to tune their textural and catalytic properties, by decreasing the pore size and the diffusivity.^153,154,159^ It is usually performed by chemical vapor deposition of alkoxysilanes, such as tetramethylorthosilicate or tetraethylorthosilicate, followed by calcination.

Activation of zeolites, upon which extraframework species are modified or removed, is crucial prior to their use as catalysts or adsorbents.^112^ Calcination at high temperatures in oxidizing (air, dry air) or inert (vacuum, nitrogen, argon) atmospheres is a frequent method to activate zeolites. If the zeolite has been synthesized in the presence of an inorganic SDA or has been subject to ion exchange, these inorganic cations lose their hydration sphere upon calcination, thus allowing for their interaction with other species. In the case of OSDA zeolites, calcination in air leads to the combustion of these organic species, thus freeing the pores. Calcination of ammonium-exchanged zeolites leads to the obtention of their acidic H-form with the corresponding release of ammonia.^41^ Additionally, calcination can result in annealing of silanol groups; i.e., connectivity defects disappear as the silanols react with each other.^160^ A specific method that allows for P-removal in zeolites which have been synthesized using a P-containing OSDA is hydrogenation at high temperature followed by calcination in air.^94^

Steaming processes involve high temperatures and an atmosphere rich in water. These promote the hydrolization of the T–O–T bonds and can have diverse effects in the final material, depending on the severity of the treatment. In the first place, the hydrolysis of the Si–O–Al (or other heteroatoms) happens, upon which the acidic properties of the material are modified. Further steaming leads to the (partial) loss of the heteroatoms present in the framework, e.g., dealumination, and the formation of silanol groups. After this, the Si–O–Si bonds start to be hydrolyzed (desilication), and the formation of mesoporosity occurs. Finally, partial or complete loss of crystallinity can happen.^141,161^

#### 2.3.4 Shaping and Structuring of Zeolitic Adsorbents for Their Industrial Use

Zeolites are mostly synthesized as a powder. Postsynthesis modification is carried out on the zeolite powder. However, prior to industrial use, this powder needs to be transformed into larger aggregates in order to improve its mechanical and physical properties with special focus on the final use.^162,163^ This transformation of the powder into aggregates is known as shaping or structuring of the adsorbent or catalyst. Shaping or structuring is important to prevent or minimize pressure drop, material loss, and erosion of equipment and can greatly affect the adsorption capacity and kinetics (see Section 3.1.2).^163−166^ In most cases, the addition of water and other inorganic and/or organic components, e.g., a binder, is needed at this stage to achieve the desired properties. The binder fills the gaps between the zeolite crystals and holds them together in the final material. It is usually an inorganic compound, such as a clay, silica, or alumina. Finally, other additives may be added, which may contribute to the plasticity of the mixture or to the porosity of the final material. There are different ways to process a powder into a more conveniently shaped or structured material:

- Granulation leads to the gradual formation of beads or pellets from the powder by controlled addition of water in the form of either droplets or a spray. The resulting beads are usually small (*d* < 5 mm) and spherical.
- Extrusion can be used to produce adsorbent pieces of different shapes. The powder is mixed with water to form a paste which is then placed inside the extruder; applying pressure forces the paste through a die of the desired shape and size. This way, polyhedral pieces with axial symmetry can be obtained (simplest case is cylinders). Extrudates that are intended for use as a single piece are called monoliths. Monoliths usually are designed to have macroscopic holes in the direction of flow which allow for fast mass transfer and reduced pressure drop.
- 3D printing is a relatively new technique which can give rise to pieces with virtually any shape.^167^ It is still mostly used at a laboratory scale, but there are some commercial providers.^168^

After shaping, the material is usually dried and calcined to remove water and labile components and to achieve the final mechanical properties.^162^ Binder content in the final material usually ranges from 5 to 30%, and a compromise must be sought between optimal mechanical properties and adsorption properties, as larger amounts of binder tend to make the material more resistant to abrasion but also less porous and thus more prone to present diffusional resistances. Binderless zeolitic adsorbents can also be produced. In this case, an “intermediate” binder is used to produce shaped adsorbent particles, which are then submitted to further treatment, e.g., hydrothermal or thermal, after which the resulting shaped material consists only of zeolite. These binderless particles tend to present less problems related to reduced adsorption capacity and kinetics.

### 2.4 Properties and Applications

Zeolites and related materials present high thermal and moderate to high chemical stabilities.^16,55^ In general terms, traditional aluminosilicate zeolites are thermally stable up to 700 °C, can be dissolved in acids and strong bases, and partially retain their crystallinity upon steaming at high temperature.^153^ Specifically, steaming at high temperatures has been used as a postsynthetic treatment to remove aluminum from the framework, increase its stability, and modify defect distribution, the most known and illustrative case of this being the development of a fluid catalytic cracking (FCC) catalyst Ultra Stabilized Y zeolite (USY).^161^ It is common that high- and pure-silica materials present even larger thermal (up to 1300 °C, relatively close to the melting point of quartz, i.e., 1713 °C^89^) and chemical stabilities (only soluble in hydrofluoric acid and concentrated strong bases). AlPOs are somewhat less thermally stable than zeolites, retaining their crystallinity at temperatures up to 1000 °C and up to 600 °C in a moist atmosphere.^55^ SAPOs tend to be moisture sensitive and slowly collapse if exposed to ambient moisture after long periods of time. In the absence of water, however, their stabilities resemble those of AlPOs. It is frequent that AlPOs and SAPOs undergo changes of structure upon hydration.^169−171^ The effect of mechanical stress on zeolites, e.g., by excessive grinding, can lead to a partial or even total loss of crystallinity.^172,173^

The most important property of zeolites, and the one on which their applicability as catalysts, adsorbents, and ion exchangers depends, is their structural porosity. Closely related to this feature, their narrow pore size distribution, i.e., very regular pore sizes, makes them useful for applications, in which size or shape selectivity is involved.^174,175^ Furthermore, their chemical properties can be tailored by synthetic or postsynthetic procedures for specific applications. When extraframework species present in their pores possess acid–base or redox properties, these are transferred to the containing zeolite to a greater or lesser extent. Below, a brief review of interesting applications and the underlying properties of zeolites is provided. Probably due to their early commercial availability, zeolites of type LTA (Linde Type A, includes zeolites 3A, 4A, and 5A) and FAU (faujasite, includes zeolites X and Y) are the most frequently addressed ones in all types of applications.

#### 2.4.1 Ion Exchangers

As mentioned in Section 2.1, the first industrial application of zeolites was as ion exchangers for water softening in laundry compositions,^24−26^ which still remains one of their major uses. In the 1950s, zeolites A, X, chabazite, mordenite, and others were tested for their ion-exchange properties.^176−178^ Depending on their pore size, these materials can act as ion exchangers for diverse cations. Logically, if the cation’s size (may also include its hydration shell) is larger than the pore opening, the exchange will not be possible to a great extent. This size exclusion together with the different affinities of ions when using zeolites as exchangers can allow for ion separation and, more specifically, ion sieving.^85,179^ For instance, zeolite 4A (sodium form of zeolite A) proved useful for the separation of Ni^2+^ and Co^2+^ cations from an aqueous solution, in which the cobalt is preferably exchanged.^178^

Since then, a great number of ion-exchange isotherms and selectivities of natural and synthetic zeolites with ANA, CHA, HEU, EDI, ERI, FAU, FER, GIS, KFI, LAU, LTA, MER, MFI, MOR, PHI, SCO, and STI structures have been determined and were reviewed by Dyer in 2007.^179^ The general conclusions on ion-exchange affinities are as follows:

- High silica zeolites tend to prefer cations with low charge density (large and monovalent), while low silica zeolites prefer cations with high charge density (small and multivalent).
- Cations that have high heats of hydration, such as Li^+^ or Mg^2+^, tend to present slow exchange kinetics.
- Other cations are usually preferred over transition metal cations (depends on the material).

It must be noted that ion-exchange isotherm measurements face an important problem when dealing with dilute ion solutions and low silica zeolites. Introducing sodium-exchanged A, X, or Y zeolites into pure water will cause an almost immediate alkalinization of the aqueous phase due to the slow exchange of sodium cations with hydronium cations,^180,181^ as follows from
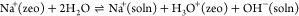
The initial increase in the pH is followed by a slow decrease, as the framework undergoes hydrolysis and part of the hydronium ions are released. At low electrolyte concentrations, and especially at low pH values, this effect will be important, and the ion-exchange properties of the material may be difficult to determine.

The use of zeolites as ion exchangers for industrial applications has been reviewed by several authors.^85,179,182−184^ Zeolite 4A has been used since the late 70s as a component in laundry detergents, replacing phosphates in their function as water softeners and thus avoiding the environmental hazard of these, i.e., eutrophication.^185^ A synthetic zeolite with GIS structure showing better performance than zeolite 4A was commercialized in 1994 for the same application.^186,187^ Natural zeolites, more specifically clinoptilolite, have been widely used for ammonium removal from water. Heavy metal cation removal from water and wastewater using zeolites has been reported as well, with clinoptilolite being again the most frequently addressed material. Furthermore, the use of zeolites in radioactive ion removal from waste streams has been known since the 1960s, when zeolite 4A was demonstrated to be highly selective toward radioactive strontium exchange.^178,188^ Natural zeolites chabazite and clinoptilolite and synthethic zeolites with CHA, FAU, and LTA structures have been used for the mitigation of the effects of nuclear accidents or the presence of radioactive waste and, more specifically, for removal of radioactive cesium.^179,189,190^

It must be noted that the use of zeolites for water treatment purposes may involve processes other than ion exchange, such as filtration, surface precipitation, or adsorption.^191^ This allows for the removal of other contaminants different than cations, such as particulate matter, anions (F^–^),^192^ or organic contaminants.

#### 2.4.2 Catalysts

Industrial application of zeolites in catalysis was first envisaged by the Union Carbide zeolite research group in the 1950s. In 1954, Milton and Breck studied the use of partially H^+^-exchanged X zeolite for the cracking of hydrocarbons and discovered it was much more active than the existing silica–alumina catalysts.^49^ That same year, they developed methods for metal dispersion in A, X, and Y zeolites and performed catalytic tests on the resulting materials.^193−197^ Shortly thereafter, and persuaded by Milton and co-workers, researchers in other companies started studying zeolites for their potential use as catalysts. In 1959, zeolite Y (FAU structure, Si/Al ≈ 2.4) was commercialized as an acid catalyst for isomerization and cracking processes by Union Carbide.^15,198−200^ In the coming years, other companies stepped in on this research field, such as Socony Mobil Oil Company, USA, and started producing their own zeolite-containing catalysts.^201,202^ Soon zeolite cracking catalysts were implemented instead of the old amorphous silica–alumina catalysts in every refinery.

Since then, zeolites have been used as catalysts in a wide variety of industrial processes, especially in oil refining and petrochemistry and processes at their interface. Zeolites with MFI, FAU, and MOR structures are the ones that have found more application niches.^203^ A description of some of the most important examples is provided below.^175,204−208^

**Figure 5:**
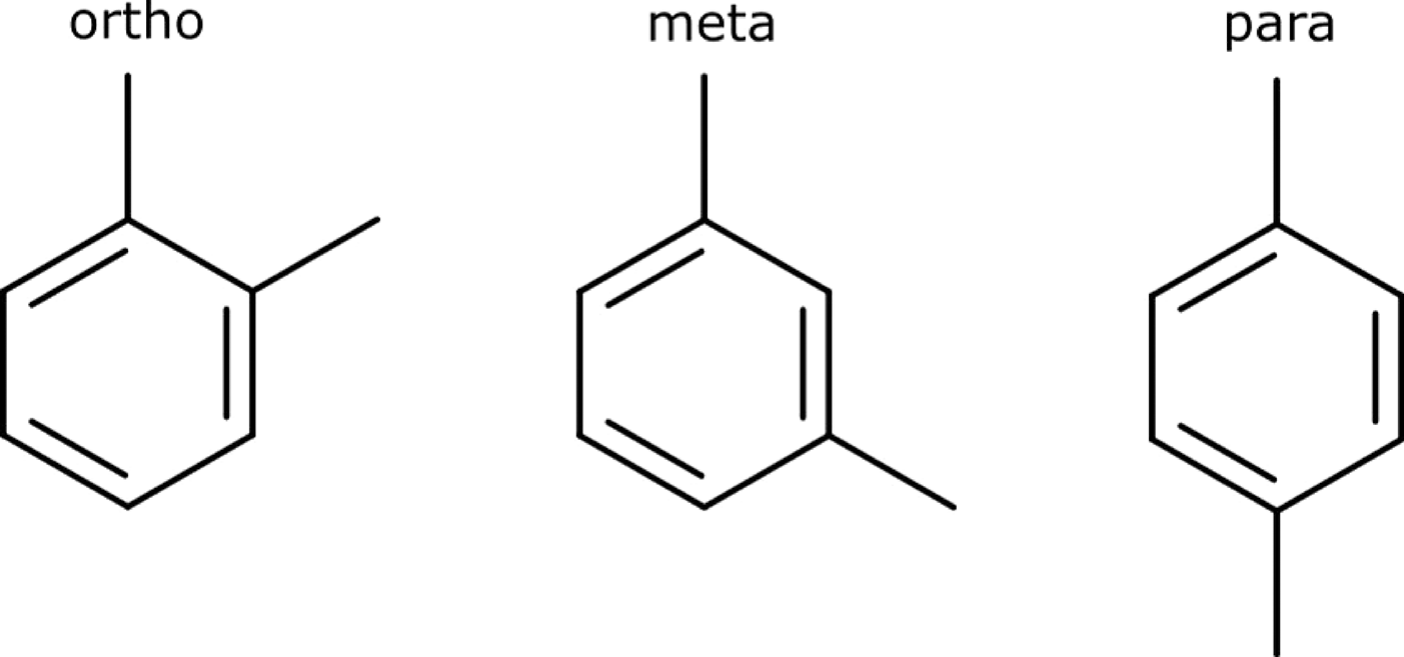
Xylene isomers. From left to right: *o*-xylene, *m*-xylene, and *p*-xylene.

**Figure 6:**
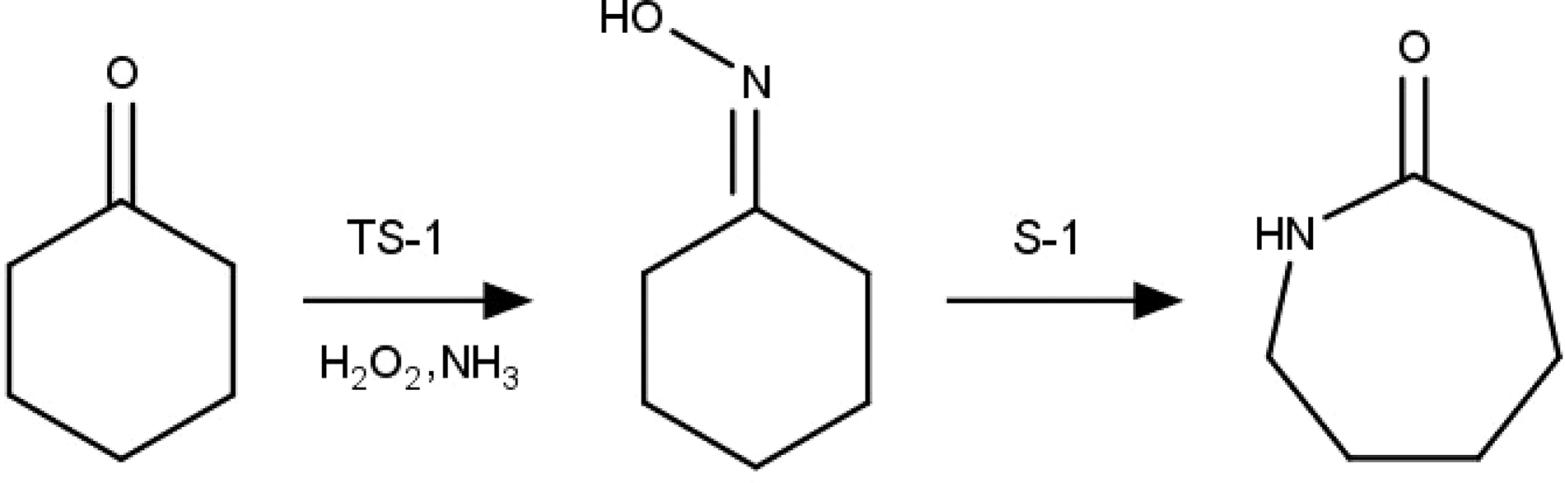
Reaction scheme for the production of ε-caprolactam using MFI-structured catalysts.

- Oil refining.
  Fluid catalytic cracking (FCC) is a process used for the production of gasoline from heavy oil fractions.^209^ Zeolites with FAU structure, more specifically Y-type rare-earth exchanged (REY) and ultrastabilized Y zeolites (USY), have been used in this application, and the latter remains the preferred catalyst for this process. The superior catalytic properties of USY as compared to amorphous silica–alumina are partly due to the presence of extraframework aluminum species, which favor the initial formation of carbenium ions, ultimately leading to an increase in the product’s octane number.^210^ Furthermore, zeolites ZSM-5 and Beta have been used as an additive in FCC catalyst compositions, as they increase the yield to light olefins and the octane number of the gasoline, respectively.
  Hydrocracking is a process in which heavy unsaturated and aromatic fractions are converted into lighter saturated compounds in the gasoline, diesel, or kerosene fractions by hydrogenation, cracking, and isomerization.^211^ Zeolite USY is used as an acid catalyst in the hydrocracking unit, along with a hydrogenation–dehydrogenation catalyst, which can be a noble metal, such as Pt or Pd, or a transition metal, such as W or Ni, depending on the sulfur content of the feed.
  Dewaxing of lubricants and fuels is a process that started using zeolites as its catalyst in the late 1960s. Acidity and shape selectivity are crucial to this process in which long-chain linear alkanes undergo cracking and/or isomerization to form branched species. In order to selectively transform the linear alkanes, medium pore zeolites have been preferably used. Industrial dewaxing processes have used catalysts based on mordenite (British Petroleum Co.),^212^ ZSM-5 (Mobil Oil Corp.),^213^ and other proprietary catalysts presumably containing SAPO-11 (Chevron),^214−216^ Beta, ZSM-22, or ZSM-23 (Mobil Oil Corp.).^217,218^
  Catalytic reforming of naphta (mainly linear paraffins in the C_6_–C_10_ fraction) produces branched alkanes and aromatics (benzene, toluene, xylenes; BTX). Reforming itself happens in the presence of hydrogen and an alumina-supported Pt–Re or Pt–Re–Sn catalyst; however, postreforming shape-selective reactions are necessary to improve the quality of the product. The first zeolitic catalyst used for this process was erionite, which allowed for selective cracking of the remaining short-chain *n*-paraffins to produce liquefied petroleum gas (LPG; mainly propane and butane).^219^ Later, ZSM-5 was introduced as the shape-selective catalyst,^220^ which also allows the entry of monobranched paraffins and benzene and toluene. The monobranched paraffins undergo cracking in the pores of ZSM-5, and the resulting olefins alkylate the aromatic species.^207,221^
  Isomerization of light straight run naphta (C_5_–C_6_ fraction) produces branched paraffins. The catalyst system needed for this reaction presents an acidic function and a hydrogenation function. Apart from superacidic chlorinated alumina and sulfated zirconia, noble-metal-supported zeolites have been used for this process, such as Pt-loaded modified mordenite^207^ and other Pt-promoted proprietary zeolitic catalysts.^204^
  Isomerization of light olefins, especially linear butenes and pentenes, produces isoamylenes (2-methyl-2-butene and 2-methyl-1-butene) and isobutene (2-methylpropene). It can be carried out in the presence of a zeolitic catalyst, more specifically modified and nonmodified ferrierites.^222−225^
  Alkylation of olefins with paraffins, mainly *n*-butene and isobutane, yielding iso-octanes is industrially carried out using liquid sulfuric or hydrofluoric acids. Several processes for alkylation wielding a zeolitic catalyst have been developed but are not operational at a large scale.^226^ Pt-supported Y zeolite^227^ and other FAU-structured materials have been reported.
  Olefin oligomerization needs propene and butenes as a starting material and yields C_6_+ iso-olefins. Phosphoric acid supported on silica was the first catalyst used for this purpose and remains the most widespread one.^228^ Some processes have been developed that use zeolitic adsorbents, such as Ni-mordenite^207,229^ and modified ZSM-5.^230−232^
- Oil refining and petrochemistry interface.
  Methanol to olefins (MTO) is a process that converts methanol into light olefins (ethene and propene). SAPO-34^233,234^ and ZSM-5^235−238^ catalysts have been commercialized for this application.
  Catalytic cracking for propene production uses primarily ZSM-5 catalysts, which favor the formation of light C_2_–C_4_ olefins upon cracking of heavier hydrocarbons.^204,207^
  Aromatization of light paraffins and olefins in the C_2_–C_8_ range produces H_2_ and BTX and is carried out in the presence of a bifunctional (acidic, dehydrogenation) catalyst. Light paraffins in the C_2_–C_4_ range can be aromatized using a zeolitic catalyst,^239^ such as Ga/HZSM-5.^240^ Hydrocarbons in the C_6_–C_8_ range can be converted into benzene, toluene, and H_2_ using an L-type zeolite.^241^
- Petrochemistry.
  *p*-Xylene (*para*-xylene, see Figure 5) is an important chemical feedstock for polyethylene terephthalate production. It can be produced by a variety of processes, most of which use ZSM-5 zeolite-based catalysts due to their shape selectivity.^242^
    Xylene isomerization processes convert *m*-xylene and *o*-xylene to *p*-xylene by using shape-selective catalysts, such as ZSM-5. Zeolites Y and Pt-loaded mordenite were used first, but the superior shape selectivity of ZSM-5 made this the catalyst of choice.^243^
    Toluene disproportionation–transalkylation processes are designed to produce benzene and xylenes (especially *p*-xylene) from toluene (along with higher aromatics). ZSM-5 and other proprietary (ATA-11, ATA-12, and ATA-21^244,245^) catalysts are used.
    Alkylation of toluene with methanol is another process for *p*-xylene production. It is carried out on modified ZSM-5 catalysts^175,242^ and other proprietary catalysts.^204^
  Alkylbenzenes (e.g., cumene, ethylbenzene) can be produced by alkylation and/or transalkylation processes of benzene and/or toluene with olefins using medium and large pore zeolitic catalysts, such as ZSM-5, modified mordenites, MCM-22, Beta, and Y, depending on the desired outcome.^175,204,246^
  ε-Caprolactam is the precursor to Nylon-6 and may be produced from cyclohexanone by ammoximation and Beckmann rearrangement. MFI-structured materials are employed as catalysts for these two steps (see Figure 6); more specifically, the ammoximation is carried out in the liquid phase with H_2_O_2_ and NH_3_ in the presence of titanium silicalite-1 (TS-1), and the Beckmann rearrangement happens in the vapor phase in the presence of silicalite-1 (S-1).^247^

#### 2.4.3 Adsorbents

The use of zeolites as adsorbents stems ultimately from their microporosity and regular pore size. The studies of Damour in 1840 and Friedel in 1896 on the reversible adsorption of molecules by zeolites were the first to shed light on the adsorption properties of these materials.^21,27^ However, it was not until McBain identified the possibility of carrying out extremely selective adsorption processes using these materials and coined the term “molecular sieves” that the way to a practical application of these was cleared.^33^ As said in Section 2.1, shortly thereafter Barrer systematically studied the adsorption of molecules of practical and theoretical interest in zeolites.^35,248^ Since then, various applications of zeolites as adsorbents for the separation of mixtures have been developed and commercialized. Due to this review’s focus being on the use of zeolites as adsorbents, a thorough review of the application of zeolites in industrial and potential adsorption and separation processes will be provided in Section 4, whereas some highlights on the characteristics and versatility of zeolites as adsorbents will be presented here.

From the point of view of the adsorption process, there are certain parameters to consider when deciding upon which adsorbent better suits the needs of the separation.^249^ The ideal adsorbent:

- is selective toward one or more components of the mixture
- presents a large (working) adsorption capacity
- is easily regenerable
- is durable and stable at relevant conditions
- is cheap
- can be shaped to achieve optimal mechanical and dynamical properties

It is difficult to find an adsorbent that fulfills all of the above characteristics for a given separation, but in any case, the best compromise needs to be sought. The selectivity in adsorptive separations (see Section 3) can be based on differences between the interactions of the different components of the mixture (thermodynamic selectivity) or on differences between the rate at which the different components are adsorbed (kinetic selectivity). The thermodynamic selectivity depends mostly on the chemical composition of the adsorbent, whereas the kinetic selectivity depends mostly on its structure and pore dimensions. Molecular sieving is an extreme case of kinetic selectivity which arises due to the very regular pore dimensions of some materials, which make them able to exclude molecules larger than the pore size. The thermal stability of zeolites together with their structural and compositional richness make them an *a priori* reasonable option for many separations. Furthermore, through postsynthetic modifications, tailored zeolitic adsorbents can be obtained.

It is not necessary to dig too deep into zeolite science to find very representative examples of tailoring of a zeolite’s adsorption properties. Zeolites 3A, 4A, and 5A, along with 13X, are the ones on which most adsorption studies have been carried out and the ones most used in industrial separations, probably due to their early commercial availability and relatively low production cost.^17,18^ Zeolite A is produced hydrothermally in its Na form and is commonly referred to as zeolite 4A, due to its pore size of 4 Å. Upon 50–70% exchange of Na^+^ per Ca^2+^, zeolite 5A (5 Å pore size) is obtained, and, analogously, K^+^-exchange of 4A yields 3A (3 Å pore size).^250^ These materials achieve to discriminate between molecules smaller than their pore size and larger ones thanks to the molecular sieving mechanism. For instance, zeolite 3A selectively adsorbs water from gas streams and liquid solvents,^12,251,252^ and zeolite 5A selectively adsorbs linear over branched hydrocarbons in the gasoline range.^253,254^ These are beautiful classic examples of how the postsynthesis modification of the pore size of a single zeolite via ion exchange allows for substantially different molecular sieve separations to be performed.

With zeolites being outstandingly shape- and size-selective adsorbents, it is not surprising that even slight changes in cation distribution or framework structure can be of high importance for their applicability as adsorbents. For instance, cation relocation coupled with a slight framework symmetry change in zeolites upon adsorption of water is a largely known phenomenon.^32,52,169,255,256^ These structure changes can affect the adsorption properties of a material, in which case they are generally referred to as gating effects.^257^ In the case of zeolites, two types of gating effects have been reported to be especially relevant, i.e., trapdoor phenomena and guest-induced framework deformation. “Trapdoor” zeolites involve a specific case of cation relocation in which the interaction of a specific molecule with an initially pore-blocking cation leads to a displacement of the cation and subsequent penetration of the molecule inside the pores. These were first described by Shang et al. in 2012, after observing remarkably high CO_2_/CH_4_ selectivities on low silica exchanged chabazites.^258^ Since then, trapdoor phenomena in zeolite adsorption have received a great deal of attention and research effort, due to the large selectivities achievable.^259−266^ These phenomena are usually susceptible to external stimuli, such as temperature,^267^ and are usually triggered by molecules that interact strongly with the extra framework cations present in the zeolite, such as water and carbon dioxide.

On the other hand, framework deformation can be as well triggered by guest species or temperature and is related to framework flexibility.^257^ Zeolites were traditionally considered as relatively rigid solids, but some adsorption and diffusion results could not be understood until flexibility was taken into account.^268−270^ Zeolite framework flexibility is the underlying reason for close-fitting molecules being able to diffuse inside the pore system.^74,271^ Furthermore, guest-induced flexibility in zeolites is a more recent discovery, beautifully exemplified by the adsorption of ethene on molecular sieve ITQ-55.^272^ Another interesting case, in which cation relocation and structure change take place simultaneously, is that of CO_2_ adsorption on RHO and related zeolites.^261,273−276^

Overall, zeolitic adsorbents present very promising properties and potential applicability for separation processes under development. The very active search for new structures that can address current problems and the key findings of the last ten years support the notion that these unique materials continue to be of high scientific and technical relevance.

## 3 Adsorption on Nanoporous Materials

Adsorption is defined as the enrichment in the concentration of molecules, atoms, or ions present in a fluid phase in the vicinity of an interface.^277^ In the case of a solid–gas or solid–liquid system, this interface is the surface of the solid. Adsorbable molecules in the fluid phase are *adsorptive* or *sorptive*; adsorbed molecules are called the *adsorbate* or *sorbate*; and the solid material receives the name of *adsorbent* or *sorbent* (when the prefix *ad-* is not present, it may be used for absorption phenomena, as well). The opposite process, in which molecules leave that surface and go back to the fluid phase, is called desorption.

Adsorbents need to possess a high specific surface area, as the maximum adsorption capacity will depend on it. Porosity increases the surface area per volume of material, thus porous materials are a common choice as adsorbents. Porous materials with pores with diameters below 100 nm are known as nanoporous materials and can be classified into different groups according to their pore size:^277^

- Microporous, with *d*_p_ < 20 Å.
- Mesoporous, with 20 Å < *d*_p_ < 500 Å.
- Macroporous, with 500 Å < *d*_p_.

There are a large number of examples of nanoporous materials, such as activated carbons, carbon molecular sieves, carbon nanomaterials, zeolites, metallosilicates, mesoporous silicas, metal–organic frameworks (MOFs), or covalent organic frameworks (COFs). Zeolites and related materials belong to the microporous materials group.

### 3.1 Basics of Adsorption

#### 3.1.1 Thermodynamics of Adsorption Processes

Adsorption phenomena are most frequently studied by measuring adsorption isotherms. In a typical experiment, the temperature is set constant, and a clean sample of adsorbent is exposed to certain values of pressure (or concentration) of the desired adsorptive/s. At each pressure *P*, equilibrium is reached, and the amount adsorbed *Q* may be calculated by the pressure drop (volumetric method) or the gain in mass (gravimetric method). Generally speaking the amount adsorbed will increase with pressure, although the shape of the isotherm may vary greatly depending on the adsorbate–adsorbent pair and the specific conditions of the experiment.

**Figure 7:**
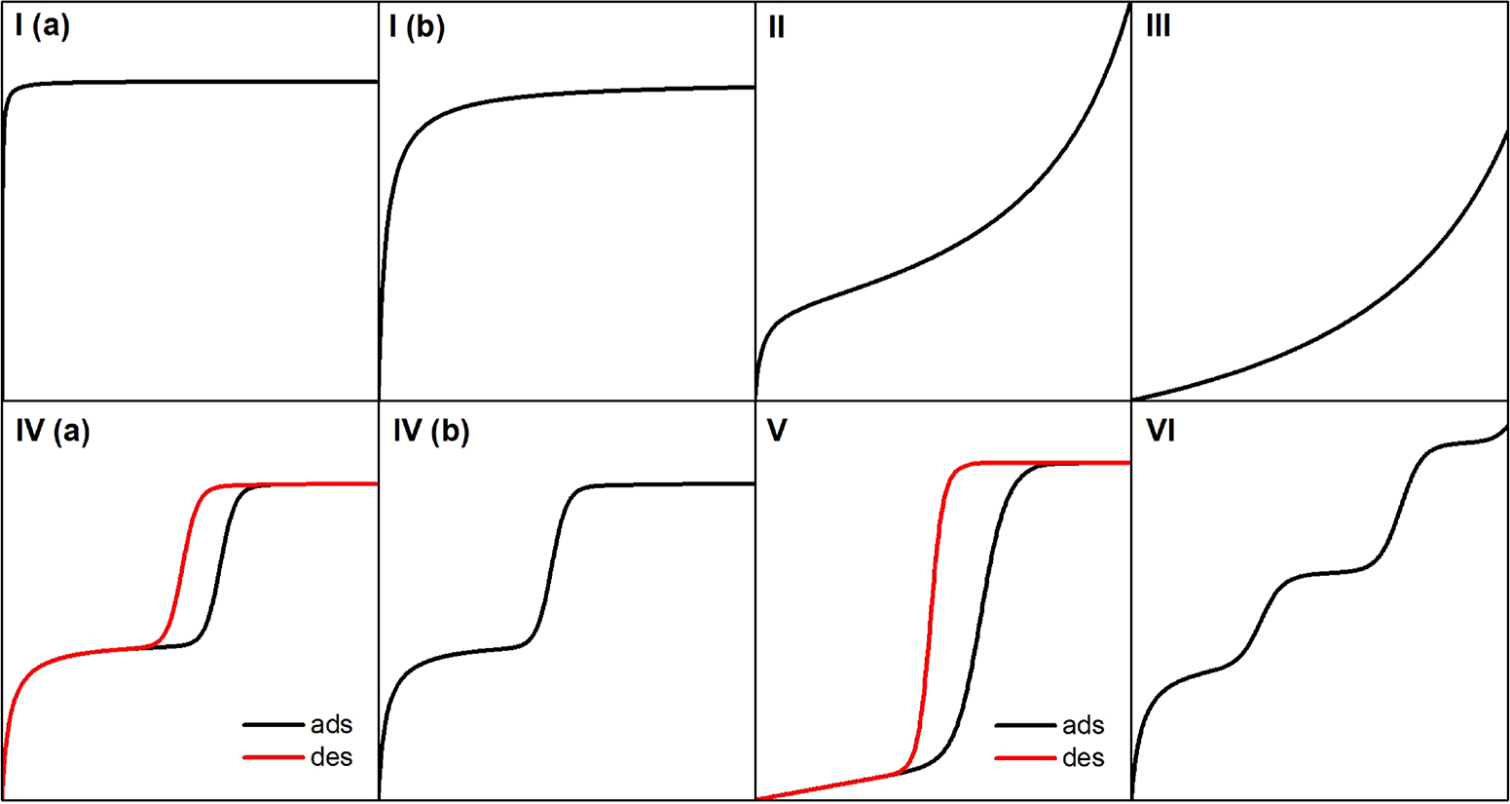
Isotherm types, according to the new IUPAC classification. The *x*-axis is relative pressure, and the *y*-axis is the adsorbed amount. In cases where a single line is depicted, adsorption and desorption are equal. Where two lines are depicted, i.e., there is a hysteresis phenomenon: red is for desorption and black for adsorption.

Years of accumulated adsorption isotherm data have allowed us to establish a classification of typical isotherm shapes (see Figure 7), which gives information on the textural properties of the solid that is being dealt with.^277^

Type I(a) and I(b) isotherms belong to microporous solids, such as zeolites. The steep low-pressure regime is due to the strong interactions that take place in the close-fitting pores of these materials. The steeper this region, the stronger the interactions. Above a certain pressure, saturation is reached, and the limited micropore space of the solid cannot take in more molecules, thus the horizontal asymptote. At pressures close to the condensation pressure, another steep increase may be seen, which is due to capillary condensation of the adsorptive outside the micropores, possibly in the space between adsorbent particles.

Type II isotherms are given by relatively weak adsorption on nonporous or macroporous adsorbents, where multilayer adsorption and capillary condensation take place. Some microporous materials present mixed features of type I and II isotherms to some extent, as the interparticle space allows for multilayer adsorption and capillary condensation. Type III isotherms belong to nonpororus or macroporous adsorbents, as well, but in this case the interaction with the adsorbate is very weak. Type IV isotherms are typical of mesoporous solids. Type IV(a) isotherms present a hysteresis loop related to capillary condensation in the pores and are given by solids in which the opening of the pore exceeds a certain value, which depends on the nature of the adsorbate. Hysteresis is a concept that refers to the case in which adsorption and desorption follow a different path in the isotherm plot. Type IV(b) isotherms belong to solids having smaller mesopores and cylindrical or conical pores with closed ends, in which capillary condensation does not result in a hysteresis phenomenon. A final plateau or inflection point is typical in this type of isotherm. Type V isotherms are seen in micro- and mesoporous adsorbents in cases where the sorbent–sorbate interaction is weak. In this sense, their low-pressure regime is similar to that of type III isotherms. At higher pressures, pore filling occurs, and the adsorbate–adsorbate interactions prevail, thus leading to a steep increase in the adsorbed amount. Hysteresis is typical in these isotherms. Type VI isotherms are given by highly uniform nonporous surfaces, in which layer by layer adsorption is distinguishable.

The underlying thermodynamics that give rise to these isotherm shapes have been reproduced via a variety of models. These models are usually specific to one or several isotherm types and are widely used for process design.^278^ As zeolites are the protagonist adsorbents of this review, some of the most relevant models used to describe type I isotherms will be briefly presented here, together with their base assumptions and shortcomings. The mathematical expressions of these models are presented in Table 1.

**Table 1:** Summary of Models Typically Used for Describing Adsorption Isotherms on Zeolites^a^

Model	Equation	Remarks	Reference
Henry	*Q* = *K*_H_*P*	Valid only at low *P*.	(280)
Langmuir	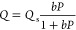	Monolayer adsorption on homogeneous solid.	(279)
Dual-Site Langmuir	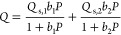	2 different types of adsorption sites.	-
Sips	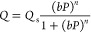	Surface heterogeneity is introduced via *n*. Gaussian-like energy distribution. Incorrect behavior at low *P*.	(281)
Unilan	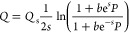	Uniform energy distribution in a certain range given by 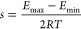	(282), (283)
Toth	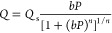	Surface heterogeneity is introduced via *n*.	(284)
Dubinin–Astakhov	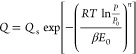	Subcritical gases. Incorrect behavior at low *P*. Homogeneous surface for *n* = 2 becomes the Dubinin–Radushkevich model.	(285), (286)
BET	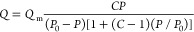	Subcritical gases. *Q*_m_ is the monolayer capacity. *C* is positive and exponentially related to the adsorption energy.	(277), (287)

a Pressures can be replaced by concentrations for liquid-phase adsorption unless otherwise noted.

In an adsorbate–adsorbent system, the simplest case that can be thought of is one where the surface presents a homogeneous energy distribution; there are no adsorbate–adsorbate interactions; and only one molecule can be adsorbed per adsorption site (monolayer adsorption). These are the basic assumptions of the Langmuir model for adsorption. This model is not precise for the description of zeolites, mainly because they are adsorbents with an energetically heterogeneous surface, but it is still a good starting point to fit type I isotherms in general. The Langmuir adsorption isotherm is presented in eq 1.^279^
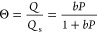
1where Θ is the fractional loading or coverage; *Q* is the adsorbed amount of an adsorbate; *Q*_s_ is the adsorption capacity at saturation; *b* is the equilibrium constant; and *P* is the pressure. At low pressure/concentration, the Langmuir isotherm becomes analogous to Henry’s law, i.e., Θ = *bP*, and thus, from fitting this model to low pressure/concentration data, the Henry constant and subsequently the adsorption enthalpy at low coverage can be obtained. The Henry constant remains one of the most important parameters for process design.^249^

Some empirical models that take the surface heterogeneity into account are derived from the Langmuir model,^280,286^ such as the Toth, Unilan, and Sips models, each presenting different approaches to simulate an energy distribution. These models tend to be a better fit for zeolite adsorption isotherms. The Toth model^284^ accounts for surface heterogeneity by means of a parameter *n* which modifies the shape of the isotherm, allowing for higher loading values at low pressure as compared to the Langmuir model. It is based on the fact that a solid presenting a heterogeneous surface will have a larger capacity at low pressure than one with a homogeneous surface. When *n* = 1, the model converges to the Langmuir model, i.e., homogeneous surface, and it behaves correctly throughout the whole pressure range. The Sips and Unilan models were both derived by Sips.^281,282^ The Sips model (also known as the Langmuir–Freundlich model) assumes a Gaussian-like distribution of the energy of the adsorption sites and presents incorrect behavior at low pressure, as it does not follow Henry’s law. The Unilan model assumes a uniform distribution of energies between two given limit values. For an infinitesimal energy range, it converges to the Langmuir model, and for wide energy distributions, it tends to the square isotherm characteristic of irreversible adsorption. It behaves correctly throughout the whole pressure range. Another frequently used way to account for surface heterogeneity is by applying the Dual-Site Langmuir model, which is a simple extension of the Langmuir model, in which two adsorption sites with different maximum loading and/or Henry constants are present. Nonetheless, the physical meaning of the parameters in cases where there are not two distinct adsorption sites is lost. The semiempirical Dubinin–Radushkevich and Dubinin–Astakhov models are applicable for condensable vapors, with the latter empirically taking into account surface heterogeneity.^285^ These models assume a micropore filling mechanism, and thus, the adsorption capacity is a function of the pore volume and the molar volume of the adsorbate. Another model that takes the condensability of the adsorbate into account and, thus, the multilayer adsorption phenomenon is the Brunauer–Emmett–Teller isotherm model.^287^ This model is only valid for a reduced pressure range, namely, 0.05–0.35 *P*/*P*_0_ (even lower in some cases),^288^ depending on the nature of the material, and is based on the assumption of a flat homogeneous surface, which is far from the reality in zeolites. However, it is one of the most widely used models for a comparison of surface area. An in-depth study of the thermodynamic implications of the models presented can be found in ref (286).

By comparing adsorption isotherms of different compounds on a specific material, a first idea of its applicability to a separation can be obtained. Ideal thermodynamic selectivities , also called pure component selectivities, can be calculated from the ratio of adsorbed amounts *Q* of different adsorbates (“a” and “b”) at a defined temperature *T* and pressure *P* (see eq 2). Alternatively, it can be defined as the ratio of Henry constants.
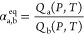
2

Obviously, pure component selectivities are a very rough approximation of the real selectivity in the case of competitive adsorption. The measurement of multicomponent isotherms is complex, and very few researchers and technicians have access to the needed equipment.^289^ Thus, models for predicting multicomponent adsorption from single-component isotherms have been developed, such as the multicomponent or extended Langmuir models,^290,291^ the ideal adsorbed solution theory (IAST),^289,292^ and other models independent or derived from the above.^291,293−295^ The Langmuir-based models and the IAST assume homogeneous surfaces, no lateral interactions between adsorbate molecules, and no size dependence, which make them poorly applicable to some systems involving adsorbents with heterogeneous surfaces or limited pore size, such as zeolites. Therefore, there is a need for said modified and new models, which introduce the effect of energy distribution, pore size distribution, or size effect. Mixture selectivities calculated from any of the mentioned models need to be contrasted with experimental data prior to their use for process design, especially if their applicability to a specific system has not been proved yet.^296^

Adsorption can be physical (physisorption) or chemical (chemisorption), depending on the strength of the interaction between the adsorbate and the adsorbent surface. The intermolecular forces that are involved in physisorption include interaction between induced or permanent dipoles and/or quadrupoles, while in chemisorption there is a change in the electronic structure of the adsorbent and the adsorbate and the formation of a chemical bond.^297^ Therefore, the absolute value of enthalpy of physisorption is generally lower (≤50 kJ/mol) than that of chemisorption (≥50 kJ/mol). It is common that an industrial adsorptive separation process using zeolites preferably involves physisorption instead of chemisorption, whereas a catalytic process involves chemisorption and further reaction. Note that adsorption is one of the necessary steps in any heterogeneous catalytic process. Physisorption phenomena are always exothermic, as the entropy decreases. This means that, at a constant pressure, the adsorbed amount will decrease with increasing temperature.

The adsorption enthalpy is defined as the energy that is released due to a specific amount of a molecule becoming adsorbed on a surface and thus has a negative value. The isosteric heat of adsorption *q*_st_ is the negative adsorption enthalpy and is positive. There are several ways to determine the experimental *q*_st_, which may be direct (calorimetry) or indirect, based on isotherm measurements at different temperatures and Clausius–Clapeyron’s eq (eq 3).
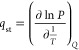
3

**Figure 8:**
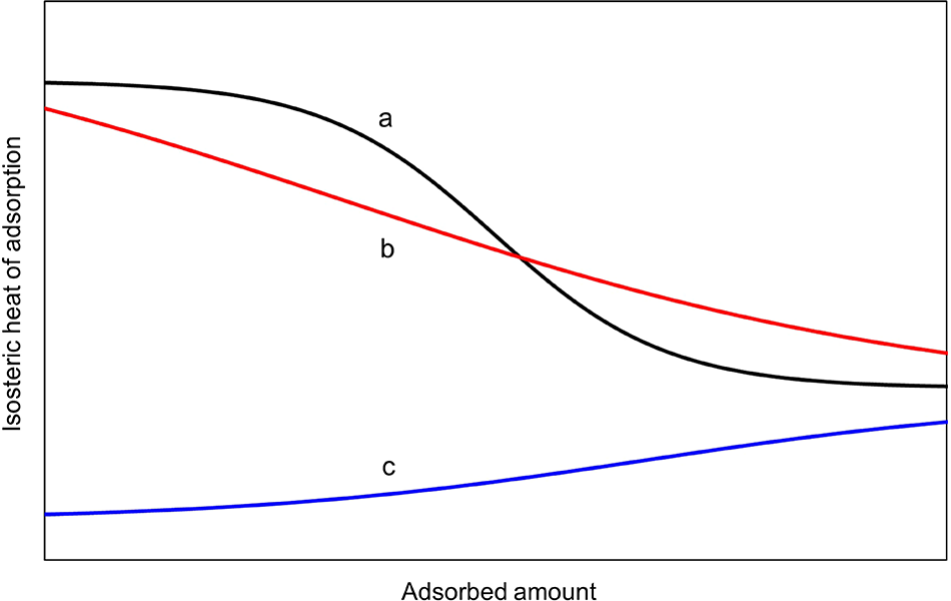
Possible trends of isosteric heat of adsorption. Trend *a* is typical of a solid with a finite number of strong adsorption sites and otherwise weak adsorption sites. Trend *b* belongs to a solid with an energetically heterogeneous surface. Trend *c* is typical of systems with weak adsorbent–adsorbate interactions.

The isosteric heat of adsorption varies with the adsorbed amount, and its trend gives information on the nature and relative strength of the interactions taking place (see Figure 8).^297,298^ Trends like *a* in Figure 8 are typical of surfaces with a finite number of relatively strong adsorption sites in which electrostatic interactions or even chemisorption takes place. When these sites are fully occupied, adsorption in other sites that present weaker interactions with the adsorbate happens and thus the drop in *q*_st_. Trends like *b*, where the *q*_st_ decreases slowly with *Q*, indicate an energetically heterogeneous surface. Trends like *c* are typical of systems where adsorbate–adsorbent interactions are weaker than adsorbate–adsorbate interactions. At larger loadings, there is an increase in the *q*_st_ due to lateral interactions of the sorbate.

Desorption, which is the opposite process of adsorption, is a necessary step in the application of solids as adsorbents or heterogeneous catalysts, as the separated or reacted species, respectively, need to be recovered. A large isosteric heat of adsorption will mean a strong sorbate–sorbent interaction and probably selective adsorption over other species, which is desirable. However, it will also involve a larger energy input (in the form of an increase in temperature or decrease in pressure; see Section 3.2) in order to desorb the adsorbed molecules, thus leading to higher energetic costs in a hypothetical separation process. Therefore, a certain compromise needs to be sought in most cases.

#### 3.1.2 Diffusion in Adsorption Processes

Diffusion of adsorptives to the surface of the adsorbent and diffusion of adsorbates inside the adsorbent are processes inherent to adsorption phenomena.^299^ These processes depend on conditions of the fluid phase, such as the temperature, pressure, and composition, and on properties of the adsorbate, such as crystal size, pore size and distribution, and surface chemistry. The mass transfer between the fluid phase and the adsorbent surface is controlled by external resistances, which vary mainly depending on the flow pattern and macroscopic shape of the adsorbent. Usually, a linear driving force model is used; i.e., the flux depends linearly on the adsorbate concentration at equilibrium with the loading at the surface. Surface resistances may hinder the diffusion of adsorbates from the surface into the pore system which can be related to stronger adsorption at the particle boundary. Finally, internal diffusional resistances control the adsorption kinetics inside the pore system and frequently represent a major contribution to adsorption kinetics.

Internal resistances vary depending on the diffusional regimes present in the system. In microporous adsorbents, diffusion takes place via successive jumps of the adsorbate between adsorption sites, i.e., regions of relatively low potential energy, and the mechanism is usually referred to as micropore, intracrystalline, or configurational diffusion. The adsorption sites are recurring in every unit cell of the zeolite, and thus the elementary adsorption steps (see below) may be characterized for a certain adsorbate–adsorbent pair. Intracrystalline diffusion can be described by several models specific to the geometry of the crystals and the nature of the system. For spherical particles, Crank’s solution to the transient diffusion equation stands (eq 4).^300^
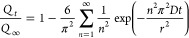
4where *Q* is the loading; *t* is the time: *n* is the number of terms of the solution; *D* is the diffusion coefficient; and *r* is the radius of the particle. The only fittable parameter is the diffusional time constant, which equals the quotient *D*/*r*^2^ and is obtained at a certain condition of temperature and pressure. In most cases, considering 20 terms of *n* is enough for fitting experimental uptake rate data and obtaining diffusional time constants. This approach is generally accepted as a good approximation to compare the diffusivities of different materials, even if they do not present spherical particle shape.

In macropores, the adsorbate–adsorbate interactions are predominant, and thus diffusion takes place via the molecular diffusion mechanism, which can be mathematically described by Fick’s first law of diffusion (eq 5).
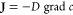
5where **J** is the flux of matter; *D* is the diffusion coefficient; and grad  *c* is the concentration gradient. The diffusion coefficient may vary with both *P* and *T*. In the mesopore range, the adsorbent–adsorbate interactions tend to be more frequent than the adsorbate–adsorbate interactions, and Knudsen diffusion is observed, in which the flux can be described by a Fickian relation with the diffusivity depending on the pore size and the temperature of the adsorbate molecules. In zeolites and other microporous solids, micropore diffusion is the most important contribution to the kinetics of adsorption, although macropore diffusion may also play a part, as the intercrystalline space resembles macroporosity. The contribution of intercrystalline diffusion will be relevant especially for adsorbents with a small crystal size.

With regard to an adsorptive separation process, diffusion is a decisive factor as to whether adsorption occurs at an acceptable rate or not. Industrial adsorbents are usually conformed into aggregates of different sizes and shapes containing not only the adsorbent but also other components, mostly inert, such as the binder (see Section 2.3.4). These shaped adsorbent particles present improved mechanical stability, handling, and pressure drop but may present different adsorptive behavior as compared to the original adsorbent material. For instance, there are numerous studies on the effect of adsorbent shaping on its adsorptive properties. The presence of inert binders, such as amorphous silica or clays, which are not porous, usually leads to a slower uptake of the final shaped adsorbent as compared to the pure adsorbent. This has been observed for zeolites, such as NaY, where the presence of bentonite binder notably decreases the diffusivity;^164^ ZSM-5, in which the presence of kaolinite as a binder was found to present a detrimental effect to diffusion, whereas alumina or silica binders did not;^165^ 5A, where the presence of a binder partially blocks the adsorption of hydrocarbons;^301^ or mordenite, where pore blockage by a silica binder was also observed.^302^ To keep the benefits of shaped adsorbents, while overcoming these transport limitations, different strategies have been developed, such as the production of binderless or hierarchically porous adsorbent particles.^166,301−303^

From the point of view of the separation mechanism, adsorption kinetics can determine how the separation is carried out. In processes that take advantage of a thermodynamic selectivity, fast adsorption kinetics, i.e., large diffusivities and mass transfer coefficients, are necessary. However, in the case that one of the components of the mixture that is to be separated diffuses much faster than others, a kinetically controlled separation may be feasible, and low diffusivities of the slower diffusing species are desirable. The extreme case, where some components of the mixture enter the pores and others are too big for entering the pores and being adsorbed, receives the name of molecular sieving. This phenomenon is very representative of zeolites, to the point that they have been referred to as molecular sieves for a long time.^11,16,49^ Kinetic selectivity and molecular sieving are achieved only on materials that present a well-defined and narrow pore size distribution.

The kinetics of adsorption can be characterized at three different levels, i.e., elementary adsorption steps and microscopic and macroscopic diffusion processes.^299^

- Elementary adsorption steps may follow many different mechanisms, depending on the specific characteristics of the sorbate–sorbent system, i.e., molecular structure, adsorbent structure, sorbate–sorbent interactions, and sorbate–sorbate interactions. They are not strictly diffusive processes, as the distances (from several Å to nm) involved are short compared to the length scales needed for the study of the overall diffusion process. In other words, a large number of elementary steps result in diffusion. Elementary adsorption steps may be assessed by molecular dynamics simulations and experimental techniques like quasi-elastic neutron scattering (QENS)^304^ and pulsed-field gradient nuclear magnetic resonance (PFG NMR),^305,306^ also considered adequate for assessing diffusion at a microscopic level. A variety of elementary adsorption steps have been observed for different types of zeolites, mostly involving jumps between specific adsorption sites. For a deeper insight into these processes, the reader may consult the extensive discussion presented by Kärger et al. in ref (299). Some generic elementary adsorption steps characteristic of intracrystalline diffusion in (cavity-like) zeolites include:
  Intracage jumps, where the adsorbate jumps from one adsorption site to another found in the same cavity. It is prone to occur more frequently when the molecule’s critical diameter is comparable to the window size and the cavity presents several adsorption sites. This motion is therefore representative of many zeolite–adsorbate systems, for instance, methane inside zeolite 4A,^307^ butane or butene isomers inside zeolite 5A,^308^ and xylenes in X-type zeolites.^309^
  Cage-to-cage jumps, where the adsorbate jumps from one adsorption site found in a cavity to another found in a neighboring cavity. This elementary adsorption step competes with intracage jumps and is the one that leads to overall diffusion. The required activation energy for these jumps to take place is comparably higher than that of intracage jumps and usually more so the smaller the size of the window relative to the molecular diameter.^310^
- Microscopic diffusion processes are studied at a scale where the adsorbate–adsorbent system is homogeneous, i.e., inside a single particle of the adsorbent, typically of the order of μm. The techniques that allow the study of microscopic diffusion processes are referred to as microscopic and are mainly based on neutron scattering, more specifically QENS,^311,312^ nuclear magnetic resonance (NMR), more specifically PFG NMR,^313−318^ and light diffraction (interference microscopy) and absorption (infrared microimaging, single-molecule fluorescence microscopy).^319−323^
- Macroscopic diffusion processes are studied at a scale that encompasses a large number of adsorbent particles and the space between them. These processes are studied by techniques such as uptake/desorption rate measurements, zero length column (ZLC) chromatography,^324−326^ or frequency response.^327,328^

In some publications on adsorption on zeolites, diffusion coefficients *D* or diffusional time constants *D*/*r*^2^ are provided. In most of these, diffusion coefficients are obtained from macroscopic measurements (uptake rate, ZLC). The ratio of diffusion coefficients of a given adsorbate pair on the corresponding material at a defined temperature and pressure gives their kinetic separation factor , also called kinetic selectivity (see eq 6).
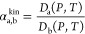
6where subindexes “a” and “b” refer to different adsorbates, with “a” usually being the fastest adsorbed component. For process design, an “effective” kinetic selectivity which takes into account also the equilibrium selectivity is preferred (see eq 7).^329^
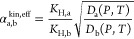
7

#### 3.1.3 Computational Methods for the Study of Adsorption

Adsorption on zeolites can be studied not only experimentally, but also via computational methods. These have gained importance over the last years, especially for the assessment of properties, situations or workloads that are not easy to study experimentally.^330^ Screening of real or theoretical adsorbents for the capture of CO_2_,^331−333^ separation of alkane isomers,^334−336^ separation of alkenes from alkenes,^337^ and other separations^338−343^ and also the study of mixtures adsorption can be approached with computational tools. Frequently, the study of thermodynamical properties and observables, such as adsorption isotherms, Henry constants, heats of adsorption, is carried out via Monte Carlo simulations, which are based on statistical mechanics.^344−346^ These can also be used to determine the pore volume and size distribution and surface area of adsorbents whose structure is known. On the other hand, molecular dynamics simulations are widely used for computing transport properties of adsorbate–adsorbent systems. Trajectories of the adsorbate in the pores can be simulated and recorded and parameters, such as mean square displacements and diffusivities, can be obtained. For a more detailed review of computational techniques for the study of adsorption on zeolites, please refer to ref (347).

### 3.2 Adsorption Processes

#### 3.2.1 Swing adsorption processes

Industrial gas adsorption processes use a technology named swing adsorption, in which the adsorbent bed is subjected to cycling conditions of pressure or temperature, thus giving rise to pressure swing adsorption (PSA) or temperature (thermal) swing adsorption (TSA). PSA is mostly used in bulk separations, where the component to be separated represents >10% of the stream to be processed, whereas TSA is preferably used in purification applications, i.e., where the component to be removed is present at concentrations <10% (usually <2%).^249,347−349^ PSA technology was developed in the 1960s^8,9^ and meant a great breakthrough, as it promoted research on adsorption processes and new adsorbents.^10,249,350^ Other variants of swing adsorption processes, including inert purge, vacuum swing adsorption (VSA), vacuum pressure swing adsorption (VPSA), electric swing adsorption (ESA), and rapid PSA (RPSA) have been developed or used in combination with typical TSA and PSA.^249,278,351^

**Figure 9:**
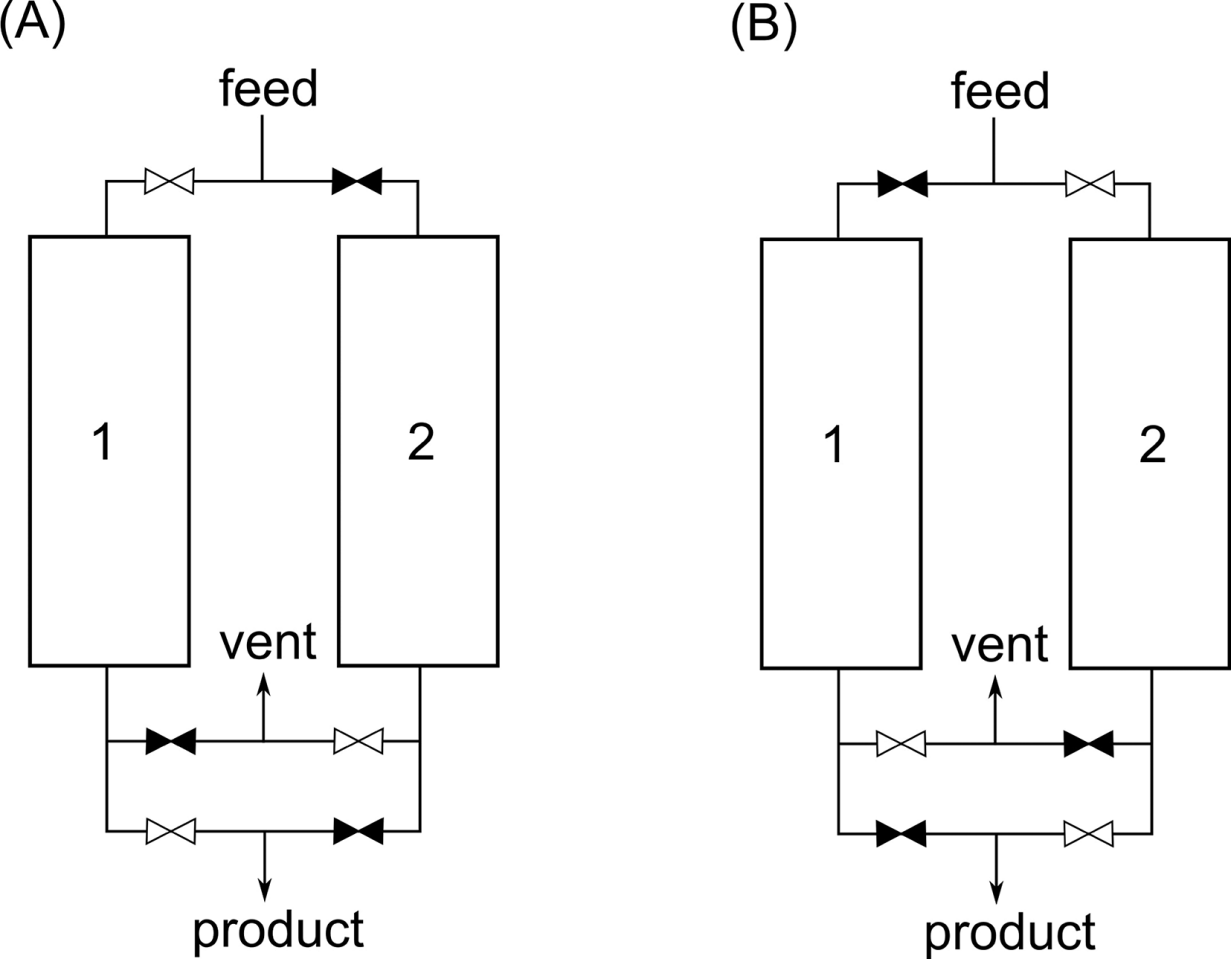
Simplified scheme of a swing adsorption process. Two adsorbent beds (rectangles 1 and 2) are connected in parallel. The pairs of opposed triangles represent valves, white when open and black when closed. In (A) the adsorption step takes place in bed 1 while bed 2 is being regenerated. In (B) the opposite situation takes place.

The conceptual scheme of a swing adsorption process is relatively simple (see Figure 9). A minimum of two adsorbent beds in parallel are necessary. Taking the case of just two parallel beds, the stream to be purified or fractionated is flown through bed no. 1 that has just been regenerated and is thus activated and ready to adsorb. Meanwhile, bed no. 2 is being subject to regeneration by either decreasing pressure (PSA, VSA, or PVSA), increasing temperature (TSA), or flowing an inert gas. A combination of desorption methods is not excluded. When bed no. 1 is saturated and bed no. 2 fully regenerated, bed no. 1 enters the regeneration step and bed no. 2 the adsorption step, thus allowing the overall process to operate continuously.^7^ The process efficiency is highly dependent on the interplay between adsorbent properties and process design, which allows for the use of different adsorbents for the same separation.^298^

Important parameters which help describe the performance of a swing adsorption process are the product purity, product recovery, and adsorbent productivity.^10^ The adsorbent productivity is the amount of feed processed per unit time and amount of adsorbent and can be expressed referring to a specific component of the mixture. The product purity refers to a certain component and equals its molar fraction (usually expressed as a percentage) in the volume-averaged product obtained throughout a certain step in the process. The product recovery also refers to a specific component and equals the amount of that component present in the product divided by the amount of that component present in the feed that has been processed.

**Figure 10:**
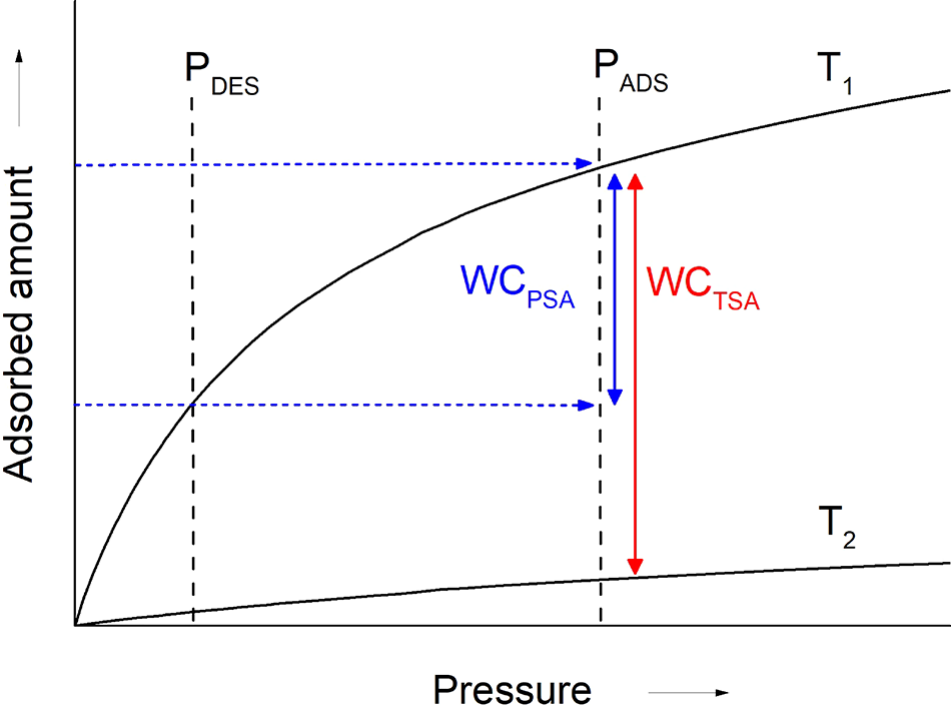
Working capacity in PSA (WC_PSA_) and TSA (WC_TSA_) processes exemplified on two hypothetical isotherms at two different temperatures on the same adsorbent. *P*_ADS_ is the pressure in the adsorption step, and *P*_DES_ is the pressure in the desorption step of a hypothetical PSA process, with *P*_ADS_ > *P*_DES_. *T*_1_ is the temperature in the adsorption step, and *T*_2_ is the temperature in the desorption step of a hypothetical TSA process, with *T*_1_ < *T*_2_. WC_PSA_ is calculated as the difference in the adsorbed amounts between *P*_ADS_ and *P*_DES_, and WC_TSA_ is calculated as the difference in the adsorbed amounts between *T*_1_ and *T*_2_.

Another important parameter in the selection of the adsorbent for a swing adsorption process is its working capacity, which is defined as the difference between the adsorbed amount at the end of the adsorption step and the adsorbed amount at the end of the desorption step and can be estimated from its adsorption isotherms. For TSA and PSA processes, a simplified graphical explanation of the working capacity is provided in Figure 10.

As can be seen, the working capacity depends not only on the equilibrium adsorption capacity of the adsorbent but also on the isotherm shape. High adsorption capacities are desired, as they will decrease the required quantity of adsorbent. Isotherms with a moderate affinity toward the adsorbate (neither too steep nor too flat) will also favor a large working capacity.

#### 3.2.2 Simulated Moving Bed Adsorption

Adsorptive separation processes may be carried out in the liquid phase as well. Techniques, such as continuous countercurrent operation (also referred to as true moving bed (TMB)) and simulated moving bed (SMB) present the highest efficiencies.^7^ Simulated moving bed adsorption overcomes some of the technical disadvantages of TMB by not involving motion of the adsorbent particles but instead a switching of inlet and outlet ports controlled by a rotary valve patented by Broughton and Gerhold for UOP in 1957.^352^ Some of the separations relevant to this review use SMB technology and zeolites as the adsorbent, such as the separation of normal from isoparaffins or the separation of xylenes.^7^

**Figure 11:**
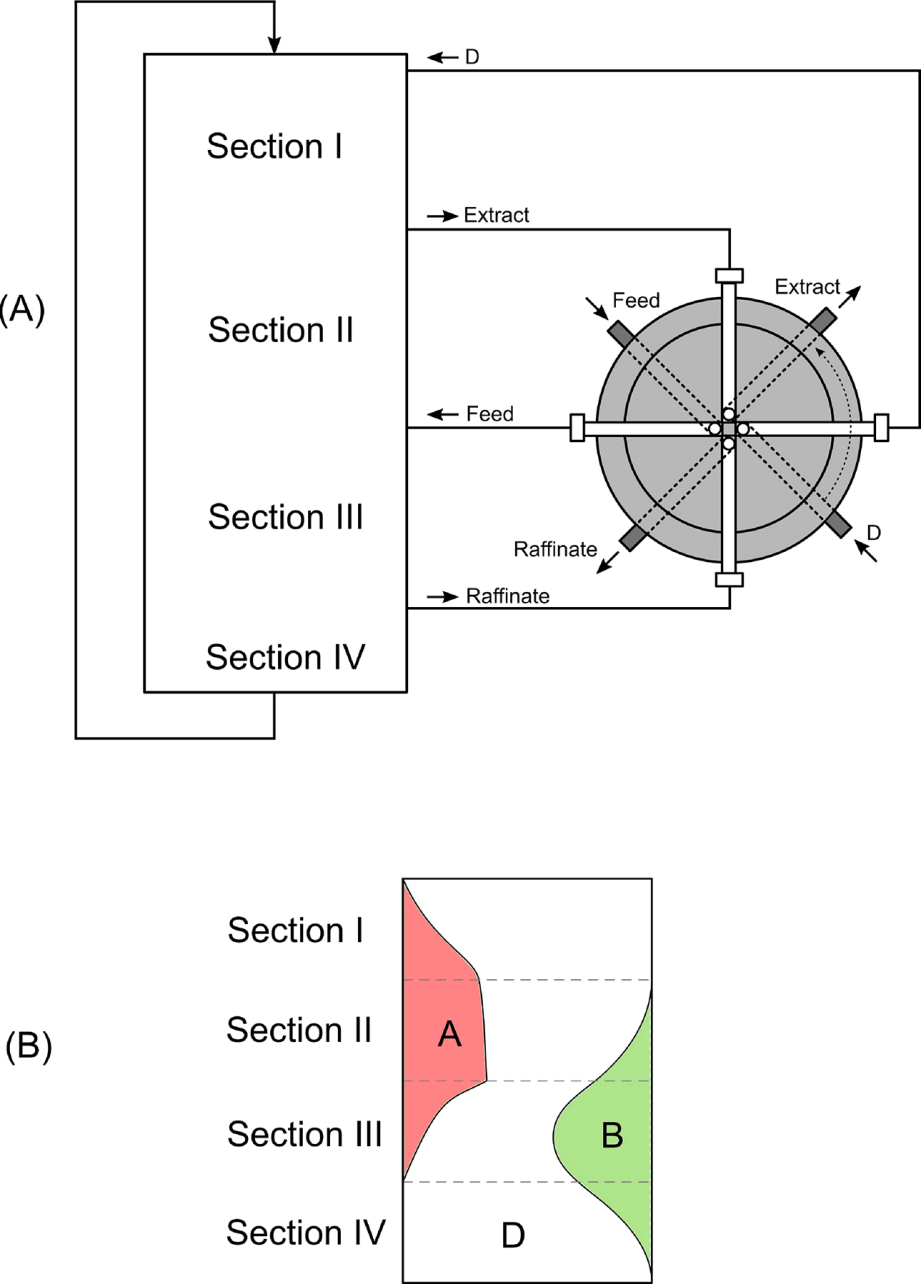
Simplified scheme of a simulated moving bed process. In (A), the layout of the process together with the rotary valve and the sections is presented. The rotary valve, the inlets of diluent and feed, and outlets of extract and raffinate are the moving parts of the system (gray). The discontinuous arrow indicates the direction of the rotation. The continuous arrows indicate the direction of the flow. In (B) the composition profile of the liquid phase is depicted.

A simplified scheme of an SMB process is depicted in Figure 11. SMB systems involve the use of a diluent/eluent (D) that may or may not be present in the feed and can act as a desorbent that displaces some of the compounds to be separated, while being displaced by others. Later, D is separated from the mixture by simple distillation and recycled. SMB systems are divided into sections, conceptually a minimum of 4.^283^ In a case where two adsorbates A and B are separated using SMB techniques and the strength of adsorption decreases according to A > D > B, the sections are

- Section I, where desorption of A takes place, is found between the inlet of the pure eluent D and the outlet of the extract (A+D). The incoming eluent D displaces A, and part of this A-rich extract leaves the adsorption bed. The fluid phase flowing into the next section contains mostly D, but also A.
- Section II, where desorption of B takes place, is found between the outlet of the extract (A+D) and the inlet of the feed (A+B). Component B is displaced by A and D, both of which adsorb more strongly. The fluid phase flowing into the next section contains the three components, A, B, and D.
- Section III, where A is adsorbed and B and D are displaced, is found between the feed inlet and the outlet of the raffinate (B+D). The fluid phase flowing into the next section contains B and D, and part of it is withdrawn as the B-rich raffinate.
- Section IV is found between the outlet of raffinate (B+D) and the inlet of additional eluent D. The fluid phase flowing into the next section contains mostly D.

The direction of flow of the liquid phase is coupled with the rotation of the inlet and outlet ports and goes from Section I to Section IV. The composition of the liquid phase is depicted in Figure 11B.

#### 3.2.3 Laboratory-Scale Study of Adsorption Processes: Breakthrough Experiments

Breakthrough experiments are a powerful tool for researchers, as they can provide information on the adsorbent–adsorbate system at conditions close to the industrial case. The separation of multicomponent mixtures can be studied, and insight on relevant thermodynamic and even kinetic parameters of the system can be calculated.

**Figure 12:**
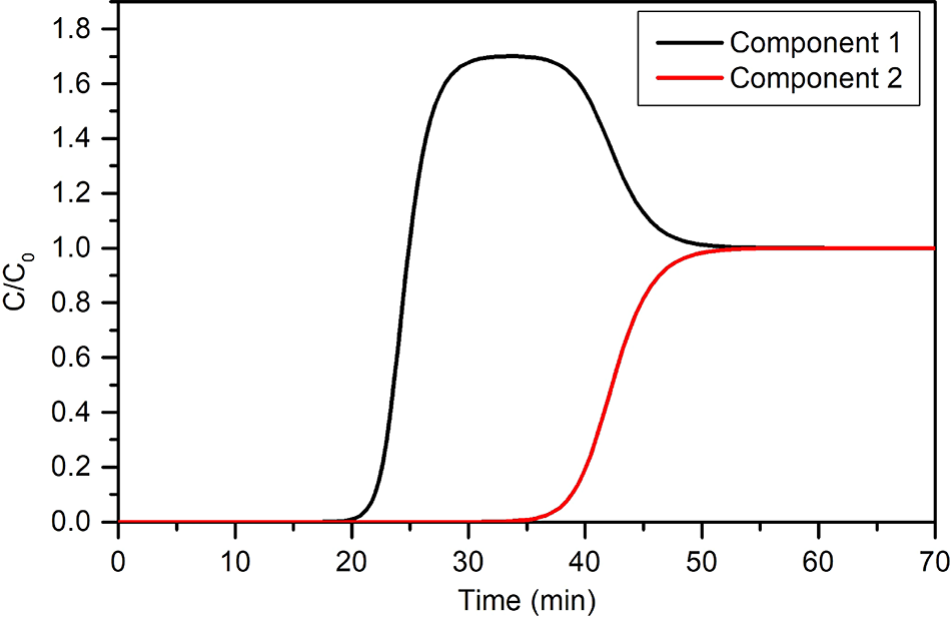
Example of a breakthrough profile of a binary mixture of compounds on an adsorbent that preferably adsorbs compound 2.

In these experiments, the adsorbent is placed in a fixed bed or column, and after activation under inert gas flow and/or at high temperature, it is exposed to a flow of an adsorptive or mixture of adsorptives. The concentration/molar flow at the exit of the column is recorded against time (see Figure 12). From the concentration/molar flow profile, thermodynamic and kinetic parameters can be obtained.

The adsorbed amount can be calculated by applying mass balance.^353,354^ Real mixture selectivities can be calculated according to eq 8:
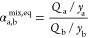
8where *y*_*i*_ is the molar fraction of component “*i*” in the fluid phase.

## 4 Chemical Separations by Zeolites

Separation processes are essential in the chemical industry, as many valuable compounds need to be extracted or purified from mixtures.^1,7^ In order to separate the components of a mixture, differences in their physical and/or chemical properties are exploited, and depending on which property the separation is based on, various types of processes/techniques are distinguished, such as distillation, extraction, crystallization, absorption, adsorption, and membrane separations.

Adsorption-based separation processes can rely on different mechanisms to achieve selectivity:

- Thermodynamic separations are performed at equilibrium, and their effectiveness relies on differences in the interaction strength of the adsorbates.
- Kinetic separations are performed away from equilibrium, and their effectiveness relies on differences in the adsorption rate of the adsorbates.
- Molecular sieving separations are exclusive to nanoporous adsorbents and imply size and/or shape exclusion of some components of the mixture from the pores. It can be understood as an extreme case of kinetic separation.

The separations relevant to this review are directed toward separating fluid mixtures. The compounds involved in these separations and relevant properties thereof are listed in Table 2. In the following sections, the state of the art of these separations will be presented and briefly reviewed with special focus on the role of zeolites as adsorbents.

**Table 2:** Relevant Properties of Adsorbates Mentioned in This Work^14,54,355−364^

	*T*_m_	*T*_b_	*d*_kin_	Polarizability	Dipole moment	Quadrupole moment
Molecule	(K)	(K)	(Å)	(10^–25^ cm^3^)	(10^–18^ esu cm)	(10^–26^ esu cm^2^)
H_2_	18.6	20.3	2.83–2.89	8.042	0	0.662
D_2_	13.8	23.6	2.83–2.89	7.954	0	–
H_2_O	273.1	373.2	2.64	14.5	1.8546	–
N_2_	63.1	77.4	3.64–3.80	17.403	0	1.52
O_2_	54.4	90.2	3.47	15.812	0	0.39
CO	68.1	81.7	3.69	19.5	0.1098	2.50
CO_2_	216.6	216.6	3.30	29.11	0	4.3
CH_4_	90.7	111.2	3.76	25.93	0	0
C_2_H_4_	104.0	169.4	4.16	42.52	0	1.50
C_2_H_6_	90.35	184.6	4.44	44.3–44.7	0	0.65
Ethanol	159.1	351.8	4.53	51.1–54.1	1.69	–
C_3_H_6_	87.9	225.5	4.67	62.6	0.366	–
C_3_H_8_	91.45	231.0	4.30–5.12	62.9–63.7	0.084	–
Acetone	178.5	329.2	4.60–4.79^a^	63.3–64.0	2.88	–
*n*-Butane	134.8	272.7	4.69	82	0.05	–
Isobutane	113.5	261.3	5.28	81.4–82.9	0.132	–
1-Butene	88.8	266.9	4.46	81	0.36–0.44	–
*cis*-2-Butene	133.9	276.9	4.2–4.94	82	0.3	–
*trans*-2-Butene	167.3	274.0	4.3–4.6	81.82	0	–
1,3-Butadiene	164.3	268.6	4.31–5.2	86.4	0	–
Isobutene	132.5	266.3	4.84	80	0.5	–
1-Butanol	183.3	390.6	4.63^a^	–	1.65	–
*n*-Pentane	143.3	309.2	4.50	99.9	0	–
Isopentane	113.3	301.0	5.0	–	0.13	–
Neopentane	256.6	282.7	6.2–6.46	102.0	0	–
*n*-Hexane	177.8	341.9	4.3	119	0	–
2-Methylpentane	119.5	334.0	5.5	–	0.1	–
2,3-Dimethylbutane	173.3	331.2	6.2^b^	–	–	–
2,2-Dimethylbutane	144.3	322.9	6.3	–	–	–
*n*-Heptane	182.6	371.6	4.3	136.1	0	–
3-Methylhexane	154.9	365.0	5.9^b^	–	–	–
2,3-Dimethylpentane	82.6	362.9	6.2^b^	–	–	–
2,4-Dimethylpentane	153.9	353.7	5.8^b^	–	–	–
*m*-Xylene	225.3	412.3	6.8^c^, 7.1^d^	142	0.37	–
*o*-Xylene	248.0	417.6	6.8^c^, 7.4^d^	145	0.64	–
*p*-Xylene	286.4	411.5	5.8^c^, 6.7^d^	143	0.1	–
Ethylbenzene	178.18	409.4	5.8^c^, 6.7^d^	142	0.59	–

a Critical diameter from ref (359).

b Critical molecular w-h parameter from ref (361).

c Taken from ref (355).

d Taken from ref (14).

### 4.1 Purification of Hydrogen

Hydrogen is primarily (>95%) produced in refineries, as a major component in steam methane reforming off-gas and refinery off-gas (SMROG and ROG, respectively).^365,366^ The compositions of these streams is as follows:

- SMROG per se consists of a mixture of CO and H_2_, i.e., syngas (see reaction 9), and can be subjected to a water–gas shift reaction process (WGS, see reaction 10) to maximize the yield to H_2_ and to decrease CO concentration for its further use in other processes.
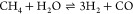
9
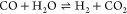
10The resulting product, and the one on which the separation is performed, typically contains 70−80% H_2_, 15−25% CO_2_, 3−6% CH_4_, 1−3% CO, and trace N_2_ and is saturated with H_2_O.^367^ The total pressure of this stream is frequently around 30 bar.^368^ Note that it is equivalent to the precombustion stream mentioned in Section 4.6.
- ROG typically contains 65–90% H_2_, 3–20% CH_4_, 4–8% C_2_H_6_, 1–3% C_3_H_8_, and lesser amounts (<0.5%) of C_4+_ hydrocarbons and is saturated with H_2_O.

The separation of the components of these mixtures is mainly directed toward producing a highly pure (>98%) H_2_ product; however, it may also be optimized to produce ammonia synthesis gas (3:1 mixture of H_2_ and N_2_).^369,370^ Additionally, the process can be designed to produce a secondary product stream containing >99% CO_2_ for its sequestration or use (CCS, see Section 4.6.2). The waste stream is frequently used as fuel for its calorific value.

The purification of hydrogen from SMROG or ROG is carried out by different means, depending on the desired product composition and purity and the intended use of the waste stream. The two types of technology that have been implemented industrially are PSA and membranes.

PSA technology is used in 85% of the hydrogen production facilities globally.^367^ PSA units use multiple columns (4 to 12) to achieve high product purities (>99.999%)^371^ and are based on the selective adsorption of the other components of the mixture, as H_2_ tends to interact poorly with the adsorbents used (H_2_ tends to interact relatively strongly with noble and transition metals and less so with other inorganic moieties^156,372−374^). Processes directed to producing only H_2_ frequently use various adsorbents in different layers in the same bed, in order to optimize the adsorption–desorption cycle. Examples of layered beds include combinations of activated carbon and zeolite 5A,^375^ activated carbon and zeolites X and Y,^376^ or activated carbon and silica gel.^377^ Processes directed to the obtention of both H_2_ and CO_2_ use combinations of adsorbents in different beds, such as activated carbon and zeolites,^378^ or add systems for CO_2_ capture prior to or after the primary H_2_ PSA.^379^ To date, zeolite 5A still remains the most competitive adsorbent for this application.^380,381^ The order in which the adsorbents are placed, the interplay between the adsorbents, and the interplay between adsorbents and process design are crucial for proper operation.

Currently, research in H_2_ purification by PSA is directed toward:^367^

- Developing RPSA (rapid PSA) processes that allow cost reduction.
- Improving the mass transfer coefficients in current and potential adsorbents by shaping them into monoliths and sheets.
- Sorption-enhanced SMR processes, in which CO_2_ is separated from the reaction medium simultaneously to its production, thus displacing equilibrium. High-temperature adsorbents are under development for this technology.^381,382^

### 4.2 Separation of Hydrogen Isotopes

The separation of the isotopes of hydrogen, i.e., H_2_, D_2_, and T_2_, is of current interest to the industry and represents an especially challenging case of separation.^342^ Deuterium (in the form of heavy water) is used as a neutron moderator in chemical reactors, as an isotopic tracer, and for the production of deuterated chemicals and drugs.^342,383,384^ Both deuterium and tritium are raw materials for fusion energy technologies, which are under intense research.^385−387^ The production of deuterium and tritium and the removal of tritium from nuclear waste^388^ are processes that require the separation of these isotopes from mixtures or compounds containing them.

Hydrogen isotopes present very similar physical and chemical properties (see Table 2), which make their separation technically difficult and/or energy intensive.^389^ Mature technologies for the separation of hydrogen isotopes, especially deuterium from hydrogen, include cryogenic distillation and electrolysis of heavy water coupled to the Girdler–Sulfide process,^383,384,388,390,391^ both of which are highly energy demanding. Other methods that have been studied are thermal diffusion, membrane technology, adsorption, chromatography,^389,392^ combinations of chromatography and cryogenic distillation,^393^ combined electrolysis catalytic exchange,^394−396^ and quantum sieving.^384,397^

Separation-oriented adsorption studies of hydrogen isotopes on activated carbons, silicas, and zeolites have been reported since the 1930s by several authors.^398−404^ According to these studies, the heavier isotopes were more strongly adsorbed than the lighter isotopes, mainly due to their larger heats of adsorption, and no remarkable influence of the pore size was observed, not even in microporous adsorbents.^399,402,403^ This thermodynamic preference toward the heavier isotope is still of interest to researchers, and new materials with improved separation prospects are being discovered, especially MOFs^405,406^ and zeolites.^407−412^ Commercial and ion-exchanged zeolites of types A, X, and Y have been most frequently studied for this purpose, and seemingly the cations with the higher charge density lead to the largest thermodynamic selectivities. Trapdoor phenomena in zeolites have been described as well, in which a D_2_-sensitive Cs-exchanged chabazite can separate D_2_ at low concentrations.^260^

In the mid 1990s, the term “quantum sieving” was proposed by Beenakker et al. to denote the quantum effect that arises when the difference between pore size and adsorbate size is close to the de Broglie wavelength of the adsorbate (it must be noted that it is conceived mainly as a kinetic effect but may also affect equilibrium adsorption).^384,397^ Since then, numerous studies featuring different kinds of adsorbents, i.e., carbon nanomaterials and carbon molecular sieves,^389,407,413−417^ boron nitride nanomaterials,^389,418^ MOFs and COFs,^419−422^ POCs,^383^ and zeolites,^342,413,423,424^ have been carried out. In the case of zeolites and zeotypes, all of the proposed materials present small pores, i.e., 8-rings and minimum pore openings below 4.1 Å, preferably below 3.3 Å.^342^

### 4.3 Drying Applications

Zeolites are widely used in drying applications at the industrial and laboratory scale.^12^ This is not surprising, as their name ultimately stems from their ability to reversibly adsorb water.^19^ Natural and synthetic low-silica zeolites are significantly hydrophilic due to their charge dispersion, i.e., negatively charged framework and extraframework cations.^16^ Water molecules interact strongly with the zeolites’ highly polar surface thanks to their dipole and quadrupole moments.^12^ Zeolites of type A (with commercial names 3A, 4A, and 5A) and X (13X) have been widely used for the drying of gas and liquid streams, mainly in TSA processes but in the case of 13X also in PSA processes.^12,58^ Even membranes of NaA zeolite have been proposed for drying of industrially relevant streams.^425^ Their ability to selectively adsorb low concentrations of water from mixtures at temperatures above ambient has helped them replace the previously used active alumina and silica adsorbents.^18,426^ Indeed, drying is one of the earliest applications where zeolites were successfully used as adsorbents.^16,426−431^ Milton patented the use of zeolite 4A in a TSA process for drying natural gas^428^ and the use of both 5A and 13X for simultaneous drying and sweetening of natural gas.^429^ He also proposed the use of type X and A zeolites for drying vapor streams.^426,431^ Zeolite 3A, which can act as a molecular sieve toward water, has been implemented in the drying of olefins,^12,54^ thus avoiding possible oligomerization inside the pores of the adsorbent. This molecular sieving effect has proven practical, as well, for drying mixtures which contain other polar compounds, such as alcohols or even the ethanol/water azeotrope.^251,432−434^ An RPSA drying process for natural gas that uses 3A zeolite as the adsorbent has been recently patented by ExxonMobil.^435,436^ Drying of streams containing highly acidic gases has been achieved by using high-silica mordenite and chabazite, which present sufficient chemical stability toward acids.^298^ Furthermore, at a laboratory scale, beads of zeolites 3A and 4A are nonregeneratively used for drying of organic solvents.^12,251,252^

### 4.4 Air: Oxygen and Nitrogen

**Figure 13:**
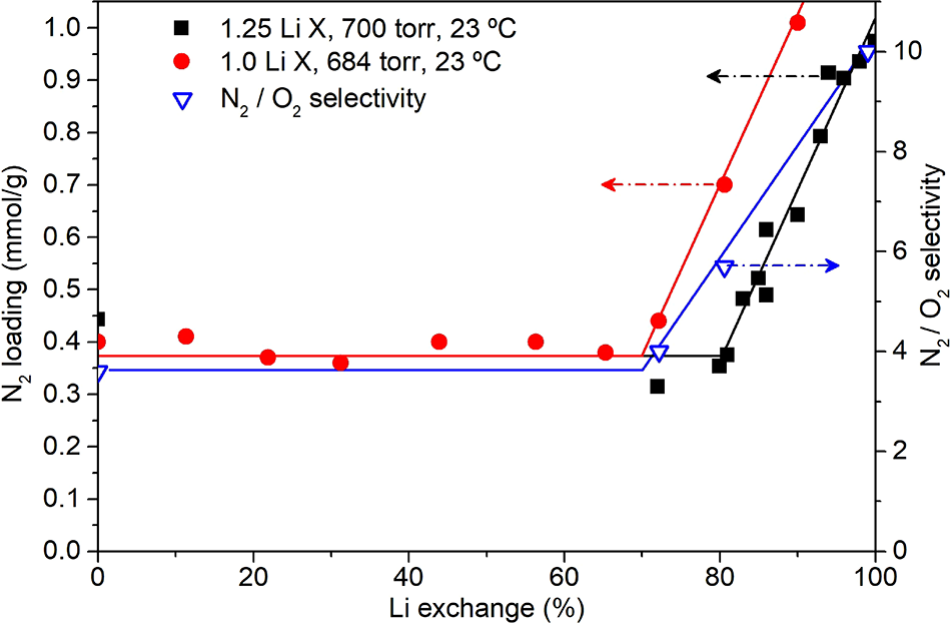
Nitrogen loading of zeolites Li(1.25)X and Li(1.0)X and selectivity of Li(1.0)X vs lithium ion exchange. Li(1.25)X stands for LiLSX with a Si/Al ratio of 1.25, and Li(1.0)X stands for LiLSX with a Si/Al ratio of 1.0. Lines are guides to the eye. Li exchange percentage refers to the total amount of extraframework cations.

The separation of oxygen from air at a large scale is carried out by cryogenic distillation. However, at smaller scales, the use of zeolite LiLSX in VPSA processes is state of the art.^4,16,249,250,437,438^ In 1959, Milton patented different type A exchanged zeolites^17^ and observed the size exclusion of N_2_ at low temperatures. He showed interest in further exploring this approach. Nonetheless, a thermodynamically controlled separation based on selective adsorption of N_2_ at close to ambient temperatures became the method of choice.^16^ Since the 1960s, different exchanged type X zeolites have been tested for carrying out this separation in PSA processes, with the focus on the obtention of pure oxygen. Three patents assigned to Union Carbide Corporation were issued in 1964 on this topic, in which the separation is achieved using zeolites of types X, Y, and L (materials with a pore size of at least 4.6 Å) at low temperatures,^439^ Sr^2+^-, Ba^2+^-, and Ni^2+^-exchanged X zeolites at ambient temperature,^440^ or Li^+^-exchanged X at ambient temperature.^437^ In the three patents, a pore size above 4 Å is pointed at as an important factor to enhance mass transfer of N_2_. The interactions of the quadrupole of N_2_ with the cations are considered the basis of the selectivity of these adsorbents. A notable effort was put into developing better adsorbents for air separation in the following years, in most cases still pulling the thread of alkali- or earth-alkali-exchanged type X zeolites.^441−443^ In 1989, Chao from UOP patented LiLSX (Lithium Low Silica X) zeolite, which presented extraordinary N_2_/O_2_ selectivity and N_2_ working capacity.^438^ Chao carried a systematic study on the Si/Al ratio and the extent of Li exchange, which allowed for his discovery. He concluded that low Si/Al ratios and Li-exchange percentages above 80% yielded materials with the best selectivities (see Figure 13). Further improvements were achieved by Kirner, who reduced the Si/Al ratio from 1.25 to 1 and the threshold of Li-exchange from 80% to 70%.^444^ This material still remains the material of choice for current processes,^4,445,446^ and its development is a beautiful example of how the properties of zeolites can be tailored for a specific application.

Some years later, another zeolite-related material, i.e., contracted Engelhard titanosilicate CTS-1 (Na,Sr-ETS-4 treated at 300–340 °C), was patented to carry out the separation of oxygen from air based on the molecular sieving of oxygen at room temperature,^447,448^ thus recovering Milton’s original idea.^17^

### 4.5 Nitrogen Removal from Natural Gas

The separation of nitrogen from methane is important in landfill gas, natural gas, and biogas processing, as nitrogen needs to be removed to increase the heating value of the mixture and meet specifications for transport through pipelines (<3%) and as liquefied natural gas (<1%).^449,450^ Currently, the most widely employed method for nitrogen removal from natural gas is cryogenic distillation, which is highly energy demanding. Alternative methods that are being researched are based on adsorption and membranes and can be selective toward either N_2_ or CH_4_.

Most known adsorbents preferentially adsorb methane over nitrogen due to the larger polarizability of the former (see Table 2).^298,451,452^ However, methane-selective pressure swing adsorption processes present the disadvantage that methane is present in these mixtures at a much higher concentration than nitrogen, and thus, the required bed size would be much larger. On the other hand, the fact that N_2_ is smaller (3.64 Å) than CH_4_ (3.76 Å) allows for a nitrogen-selective separation under kinetic control and even by molecular sieving.

Zeolite 4A was patented for this purpose by Habgood,^453^ but the hydrophilicity of this material and the high temperatures needed for its activation rendered it impractical.^454^ Natural clinoptilolites in their original and calcium-exchanged forms have been studied as adsorbents that kinetically distinguish between nitrogen and methane.^455^ Clinoptilolite in its magnesium-exchanged form was patented by Chao for its use in a PSA unit to separate N_2_ from CH_4_.^456^ Titanosilicate materials developed by Engelhard Corporation (now BASF), such as ETS-4, CTS-1, Ba-ETS-4, and Sr-ETS-4, have been demonstrated to be excellent adsorbents for this application at a small scale.^448,451,457−459^ They have been commercialized under the name Molecular Gate and consist of a mixed octahedral–tetrahedrally coordinated framework with 8-ring openings, the size of which can be tailored by ion-exchange and thermal treatment.^447,452,460^ Ion exchange can improve the thermal stability, and with it the reusability, of these titanosilicates.^459^ All the mentioned materials present minimum pore openings between 3 and 4 Å, which account for the kinetic discrimination of N_2_ from CH_4_.

### 4.6 Separation of Carbon Dioxide

Carbon dioxide (CO_2_) is a ubiquitous compound, found mainly in the gaseous state on the Earth. Its separation from mixtures with methane, nitrogen, water, hydrogen, etc., is a very active research topic.^5,58,451,461^ Mixtures of industrial interest, where CO_2_ is sought to be removed, are classified according to the reason for interest and listed below:

- Methane-containing mixtures with intended use as fuel:
  Natural gas, a fossil fuel where the main component is usually CH_4_ (30–98%), and the other components (CO_2_, light hydrocarbons, H_2_O, and H_2_S) are present in variable amounts.^450,462−464^ It can appear associated with an oil deposit or nonassociated.
  Coalbed methane, which is fossil methane found along with coal, with a methane content of 50–99%, typically above 80%, and variable amounts of CO_2_, N_2_, light hydrocarbons, H_2_S, and SO_2_.^465−468^
  Landfill gas and biogas and renewable fuels derived from fermentation of residues and biomass, which contain CH_4_ and CO_2_ as the major components and a considerable amount of N_2_ and H_2_O.^469,470^
- Hydrogen containing mixtures, such as steam methane reforming off-gas and refinery off gas (see Section 4.1).
- Mixtures where the main objective is to capture the CO_2_.
  Postcombustion streams, also called flue gases, which are the byproduct of combustion processes for energy production in general and also importantly in cement and metallurgy industries. The composition of this stream is mainly N_2_ (70–75%), CO_2_ (3–30%), H_2_O (5–7%), and residual O_2_ (3–4%), with minor amounts of CO and nitrogen and sulfur oxides.^368,471,472^ Oxyfuel technology is a special case, in which the fuel is burnt in the presence of oxygen instead of air, and thus, nitrogen is not present in the flue gases (CO_2_ content >90%^473^).
  Ambient air, where CO_2_ is only a lesser component (416 ppm in August 2021^474^).

The separation of CO_2_ from these mixtures generally uses similar principles, as, independently from the aim, they focus on retaining the CO_2_ and leaving the other components in the mixture.^449^ They even overlap in what refers to carbon dioxide capture in natural gas processing or hydrogen production.^5^ The state of the art of the mentioned separations is summarized below, except for the case of hydrogen production that can be found in Section 4.1.

#### 4.6.1 Removal of Carbon Dioxide from Methane-Rich Mixtures

Methane (CH_4_) can be obtained from fossil (natural gas, coalbed methane) or renewable (biogas and landfill gas) sources, and its major use is as a fuel, its global electric power generation share being 23% in 2018 and with expectations of growth in the coming decades.^475,476^ It also serves as a starting material in some petrochemical processes, such as methane reforming for syngas and/or hydrogen production.^477−479^

Natural gas is found in underground deposits, frequently along with oil (associated natural gas) or coal (coalbed methane). Biogas and landfill gas are produced in anaerobic digestion processes of anthropogenic waste, which take place in sewage plants (wastewater) or landfills (solid waste), respectively.^469,470^ These methane-containing gas mixtures need to be upgraded to meet specifications prior to use and/or transport. Components with no calorific value, such as CO_2_, H_2_O, and N_2_, have to be kept below specific levels in order to allow for the use of the mixture as a fuel. Furthermore, H_2_O, CO_2_, and other minor components, such as H_2_S, need to be removed to prevent corrosion and plugging problems in the processing and transportation operations.^451,469,480^ Hydrocarbons in the C2 and C3 fractions contribute positively to the heating value of the mixture and do not need to be removed generally. However, hydrocarbons longer than propane need to be separated despite their potential contribution to the calorific value of the mixture, as they can condense during the processing and cause plugging problems.^450,481^

As suggested above, the removal of carbon dioxide is central to the upgrading process, as it is frequently a major component of these mixtures (see Section 4.6), and apart from being a diluent and decreasing the heating value of the mixture, it is also a sour gas, which can cause plugging problems and corrosion.^449,450^ The state-of-the-art techniques for CO_2_ removal from natural gas include absorption in chemical, physical, or hybrid solvents, adsorption, membranes, cryogenic distillation, and methanation.^450,482^ Absorption in aqueous alkanolamines involves the formation of a carbamate upon flowing the gas through the amine solution, and it is the traditionally preferred method for large-scale facilities.^449,450^ After this process, the treated gas is saturated with water and will require drying. The amine solution needs to be regenerated to release the acid gases (CO_2_ and H_2_S), a step which is highly energy intensive. The high capital and operation costs inherent to this technology make it impracticable for small-scale facilities and remote deposits. In the search for more optimal and environmentallly friendly processes, also applicable to medium- and small-scale facilities, other separation techniques are under consideration and research. Adsorption (more specifically PSA, pressure swing adsorption) and membrane technologies have the potential to be much less energy intensive and to reduce the operation costs significantly.^449−451,463,483,484^

PSA processes for carbon dioxide separation from methane have been studied on a wide range of materials, such as zeolites, metal–organic frameworks (MOFs), carbon molecular sieves, activated carbons, porous polymers, or amine-impregnated mesoporous silica.^449,451,463,485,486^ Out of these, zeolites, titanosilicates, carbon molecular sieves, and metal-based adsorbents have found commercial application.^449^ However, the search for better adsorbents that can further improve the efficiency and economy of the process is still a very active research field.

Traditional A-, X-, and Y-type zeolites have been commercialized or patented for this separation,^463,487,488^ as well as silicalite, mordenite,^489^ natural clinoptilolite,^490^ and titanosilicate ETS-4.^451,491−494^ There are different ways that zeolitic adsorbents may be selective toward CO_2_. One is by exploiting differences in the interaction strength, i.e., the heat of adsorption, of the two adsorbates. Another possibility involves discriminating between their sizes, e.g., molecular sieving. More complex mechanisms, such as trapdoor mechanisms and guest-induced structure flexibility, are also interesting, as they usually lead to extremely high selectivities (see Section 2.4.3).

Selectivity on low silica (highly polar) zeolites, such as A, X, and Y zeolites, is achieved by the first mechanism. CO_2_ adsorbs more strongly than methane on these materials due to the electrostatic interaction of its quadrupolar moment with the extraframework cations.^483^ In some cases even chemisorption takes place, and strongly bound carbonate-like species can be formed.^495,496^ In consequence, these materials present large thermodynamic CO_2_/CH_4_ selectivities. However, this is disadvantageous for the regeneration step, as a higher energy input, i.e., higher temperatures in TSA or lower pressures in PSA processes, will be needed for desorbing the strongly adsorbed (or even chemically bonded) CO_2_. Furthermore, natural gas and the other addressed gas mixtures frequently contain a certain amount of water, which will also strongly adsorb on these highly polar (and thus hydrophilic) zeolites. It may be the case that this simultaneous removal of water and CO_2_ is intended, but this depends greatly on the specific process conditions.^429,497^ In most cases, this competitive adsorption is undesired, and it is also noteworthy that water not only will compete with CO_2_ in the adsorption process but also may favor its chemisorption and the formation of bicarbonate-like species.^495,496,498,499^

In the case of ETS-4 and related MolecularGate technology,^451,492^ the efficiency of the process is based on molecular sieving. The right pore size of the adsorbent is selected by controlled thermal treatment. In coherence with this approach, part of the current research on this separation is directed toward finding materials with a lower surface polarity, and thus a lower heat of adsorption of CO_2_, that maintain a high CO_2_/CH_4_ selectivity as well as a large working capacity thanks to their pore size and topology. These parameters can be tuned by proper selection of the structure and composition.^273,483,500,501^ Promising values of CO_2_/CH_4_ selectivity and heat of adsorption of CO_2_ have been obtained using medium-, high-, and pure-silica LTA,^501^ RHO,^273^ MWF, PWN^275,502^ (and other RHO-related structures), FAU,^483^ CHA,^503^ AEI, STT, and RRO^504^ zeolites and aluminophosphates (AlPOs) and silicoaluminophosphates (SAPOs) with CHA, RHO, AFX, AFN, and AEI structures.^152,505−508^ Some recent patents aim in this direction, as well, presenting materials, such as SSZ-45 (EEI structure), zeolite RHO, and ITQ-55.^509−511^ Out of these new-generation zeolitic adsorbents, structures featuring small pores (8-rings) stand out, as they maximize the intrinsic structural selectivity. This seems reasonable, as they present similar pore diameters (ca. 3–4 Å) than the kinetic diameters of CO_2_ (3.3 Å) and CH_4_ (3.8 Å). Note that, despite being promising, these materials are relatively expensive to obtain, due to the need of an OSDA, which hinders their commercial development.

In the case of RHO and RHO-related structures, the selectivity is enhanced by structural changes in the framework and in cation relocation upon CO_2_ adsorption.^261,263,273−275,502,512−515^ These structural changes and trapdoor effects do not take place upon CH_4_ adsorption, which leads to extremely high selectivities. Similar but more severe trapdoor effects upon CO_2_ adsorption are observed in low silica aluminosilicate CHA materials containing potassium and prepared in the absence of an OSDA.^258,262,516^ Nonetheless, said CHA materials operate poorly under the presence of water, which is a major issue for industrial implementation.^516^ Merlinoite is another zeolite which presents guest-induced flexibility upon CO_2_ adsorption and very promising CO_2_/CH_4_ selectivities.^264,517,518^ Additionally, Hong et al. have reported its OSDA-free synthesis, making it a very promising and potentially cheap CO_2_ adsorbent.^264^

Table 3 lists many of the mentioned adsorbents and includes relevant descriptors and parameters related to the adsorption of CO_2_ and its separation from CH_4_. The Si/Al ratios have been included as well as the estimated framework negative charge, in order to rationalize the different types of adsorbents as high-, medium-, or low-polarity materials.^55^ The estimated framework negative charge is a parameter introduced by our group in a previous publication^152^ in order to be able to compare zeolites with AlPOs and SAPOs, i.e., materials which are not based on silica. Values below 0.17 correspond to low polarity materials, while values above 0.33 correspond to highly polar materials. Loadings at 1 bar, working capacities between 5 and 1 bar, ideal selectivities of CO_2_ over CH_4_ at 1 and 5 bar, and isosteric heats of adsorption at low CO_2_ loadings  have been collected from the literature and are displayed in Table 3. Note that CO_2_ partial pressures at the inlet of a CO_2_ removal unit are 0.4–35 bar,^450,519^ most frequently above 1 bar. Thus, pressures from 1–10 bar are typical for the feed.^451,483,520^

**Table 3:** Zeolitic Adsorbents for CO_2_ Separation from CH_4_^a^

Material	Structure	Si/Al ratio	Estimated framework negative charge^b^	*T* (°C)	*Q*_1bar_ (mmol/g)	WC_5–1_ (mmol/g)	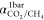	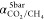	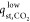 (kJ/mol)	ref·
Si-ITQ-29	LTA	inf	0	30	1.04	3.11	3.5^c^	3.2^c^	21.0	(501)
LTA-5	LTA	5	0.17	30	3.07	2.51	7.2^c^	3.5^c^	32.8	(501)
LTA-5^d^	LTA	5	0.17	30	2.90	0.70	-	20	32.8	(483)
LTA-5	LTA	5	0.17	30	3.36	2.17	7.9	3.0	32.8	(483)
5A	LTA	1	0.5	25	4.67	-	5.8	-	-	(521)
4A	LTA	1	0.5	30	4.23	0.81	3.2^c^	1.2^c^	49.0	(501)
AlPO-42	LTA	-	0	25	1.26	3.21	3.9^c^	3.7^c^	11.9	(152)
AlPO-42^d^	LTA	-	0	30	1.00	0.25	-	6.0	24.4	(483)
AlPO-42	LTA	-	0	30	1.97	2.12	4.9	3.2	24.4	(483)
Na,Cs-RHO	RHO	4.5	0.18	30	3.49	2.07	75^c^	8.8	33.0	(273)
M-DNL-6	RHO	-	<0.18	25	4.65	-	12.2^c^	-	39.0	(505)
Na-SAPO-RHO	RHO	-	<0.18	25	3.45	-	179	-	42.5	(263)
Na-SAPO-RHO^e^	RHO	-	<0.18	25	1.52	-	618^c^	-	42.5	(263)
PST-29	PWN	4.5	0.18	25	4.26	-	6.9	-	-	(502)
NaTEA-ECR-18	PAU	3.5	0.22	25	2.99	0.17	11^c^	15.6	53.0	(275)
NaTEA-ZSM-25	MWF	3.4	0.23	25	3.50	0.29	22^c^	42	65.0	(275)
Li(0.13)-ZSM-25	MWF	3.15	0.24	30	2.4	1.4	-	-	33	(515)
Li(0.13)-ZSM-25^f^	MWF	3.15	0.24	30	1.94	-	67	-	33	(515)
NaTEA-PST-20	-	3.1	0.24	25	3.17	0.73	15^c^	24	44.0	(275)
K-MER-2.3	MER	2.3	0.30	25	2.99	-	12	-	30	(264)
K_6.2_-MER-4.2	MER	4.2	0.19	25	3.74	0.66	-	-	35	(517)
K_6.2_-MER-4.2	MER	4.2	0.19	25	-	-	154^g^	-	35	(517)
13X	FAU	1.2	0.45	25	4.69	1.36	7.6	3.0	37.2	(522)
13X^h^	FAU	1.2	0.45	30	4.13	1.13	89	66	-	(520)
NaX^d^	FAU	1.2	0.45	30	4.30	0.30	-	61	37.5	(483)
NaX	FAU	1.2	0.45	30	5.60	0.97	7.7	2.8	37.5	(483)
EMC-1^d^	FAU	3.8	0.21	30	4.80	0.40	-	41	33.0	(483)
EMC-1	FAU	3.8	0.21	30	5.23	1.68	17.4	4.6	33.0	(483)
Na-USY^d^	FAU	8.6	0.10	30	1.20	0.70	-	12.0	29.3	(483)
Na-USY	FAU	8.6	0.10	30	1.73	2.15	6.2	4.0	29.3	(483)
SAPO-37^d^	FAU	-	-	30	2.80	1.00	-	14.0	31.3	(483)
SAPO-37	FAU	-	-	30	2.56	2.27	10.9	4.2	31.3	(483)
Si-SSZ-13	CHA	inf	0	25	2.24	2.38	4.0^c^	2.7^c^	25.3	(152)
Si-SSZ-13	CHA	inf	0	30	1.90	-	4.2	-	23.1	(504)
r1KCHA	CHA	1	0.5	30	1.33	0.21	15	10	48.9	(516)
r1.2KCHA	CHA	1.2	0.45	0	2.11	0.36	93^c^	33	-	(258)
r1.9KCHA	CHA	1.9	0.34	0	0.81	-	354	-	-	(262)
r1.9KCHA^i^	CHA	1.9	0.34	30	-	-	583	-	-	(262)
r1.9KCHA	CHA	1.9	0.34	30	1.49	-	105	-	-	(262)
CHA-6	CHA	5	0.17	30	4.65	1.01	3.7^c^	2.4^c^	36.6	(152)
AlPO-34	CHA	-	0	25	1.60	1.90	4.0^c^	2.7^c^	22.3	(152)
SAPO-34	CHA	-	0.15	25	2.80	1.84	5.0^c^	2.8^c^	31.5	(152)
SAPO-34	CHA	-	-	25	2.82	-	8.8	-	-	(523)
Si-SSZ-39	AEI	150	0.01	30	1.82	-	4.0	-	26.2	(504)
Si-SSZ-23	STT	inf	0	30	1.99	-	4.9	-	23.5	(504)
Si-RUB-41	RRO	inf	0	30	1.90	-	10.0	-	28.6	(504)
Rb-ZK-5^h^	KFI	3.8	0.21	30	2.30	0.70	17	8	37.0	(520)
Rb-ZK-5	KFI	3.8	0.21	30	2.67	0.80	5.5	3.4	37.0	(520)
Cs-ZK-5^h^	KFI	3.8	0.21	30	1.70	0.67	17	9	33.0	(520)
Cs-ZK-5	KFI	3.8	0.21	30	2.25	0.83	4.4	2.8	33.0	(520)
SAPO-56	AFX	-	<0.15	20	3.86	-	5.4	-	36.0	(506)

a Adsorption isotherm values have been extracted from the references where they were not explicitly displayed. Selectivities have been calculated therefrom, unless otherwise specified.

b The estimated framework negative charge gives analogous information as the Si/Al ratio and is defined in ref (152). Materials with values above 0.33 are considered to be highly polar.

c These selectivities have been directly taken from their respective references.

d Breakthrough data of a CO_2_/CH_4_ 50:50 mixture^483^ at 5 bar total pressure.

e Breakthrough data of equimolar CO_2_/CH_4_ mixture, 1 bar total pressure.^263^

f Breakthrough data of equimolar CO_2_/CH_4_ mixture, 2 bar total pressure.^515^

g Breakthrough data of 10/30/50 (CO_2_/CH_4_/He), 1 bar total prressure.^517^

h Breakthrough data of a CO_2_/CH_4_ 40:60 mixture^520^ at 5 bar total pressure.

i Breakthrough data of equimolar CO_2_/CH_4_ mixtures^262^ at 1 bar total pressure.

Data obtained from breakthrough experiments of mixtures result in systematically higher selectivities than the ones obtained from the pure-component isotherms for the same material. This could be due to competitive adsorption phenomena of CO_2_ and CH_4_ taking place. The highest selectivities come from materials that present trapdoor phenomena, especially those with RHO, PAU, and MWF structures. Zeolite 13X (or NaX) also presents very high selectivities. However, except for Na,Cs-RHO, the working capacities on most of these materials tend to be low, due to the isotherm reaching saturation at low pressures, and the heats of adsorption present relatively high values, indicating a highly energy-demanding regeneration. Lower polarity FAU, CHA, and LTA materials present good selectivities, intermediate working capacities, and moderate heats of adsorption in the range of 27–33 kJ/mol, described as optimal for a thermodynamic separation.^483^ Pure silica zeolites and AlPOs with small pores present the largest working capacities and lowest CO_2_ heats of adsorption, and some of them, such as Si-RUB-41, SAPO-34, SAPO-56, or M-DNL-6, present selectivities comparable to the ones of higher polarity materials.

#### 4.6.2 Carbon Dioxide Capture

Carbon dioxide (CO_2_) occurs from both natural and anthropogenic sources, with anthropogenic contributions (transport, industry, energy production) being by far larger than natural ones (respiration of living beings, tectonic activity). The anthropogenic CO_2_ emissions have been rapidly increasing for the last 50 years, and they surpass largely the amount of CO_2_ that the biosphere can reabsorb.^524^ Furthermore, there is a clear correlation between these greenhouse gas emissions (of which CO_2_ is the main contributor,^525^ followed by CH_4_ and N_2_O) and climate change, and therefore, there is an urgent need to mitigate their effect.

Anthropogenic carbon dioxide emissions can be prevented and countered following different strategies, such as optimizing the use of energy, reducing carbon intensity by switching to renewable energy sources, or enhancing its sequestration.^526^ Despite the great effort that is being put into the first two options, it is widely accepted that the world’s energy supply will continue to depend on fossil fuels to some degree for at least this century,^524^ and this intrinsically will lead to CO_2_ production. Thus, CO_2_ capture and sequestration (CCS) is a necessary strategy to reduce CO_2_ emissions and its concentration in the atmosphere and to mitigate climate change.^5^

CCS technologies have been implemented in different industries and deal with mixtures of diverse nature, such as natural gas, steam methane reforming off-gas (precombustion stream), flue gases (postcombustion stream), and ambient air. A total of 19 large-scale CCS facilities were in operation in 2019, out of which 10 have been implemented in natural gas upgrading, 3 in hydrogen production (precombustion), 2 in fertilizer production, and 2 in power generation (postcombustion). It is noteworthy that most CCS operating facilities have been implemented in industries where CO_2_ removal needs to be performed anyway.^5,527^ Still, a large effort needs to be put into the development and deployment of more CCS facilities.^5^

At the present time, and similarly to the case of natural gas processing, the most mature CO_2_ removal technique for postcombustion streams is chemical absorption with aqueous amines.^5,527−529^ The flue gas of processes that use oxy-fuel technology consists mostly only of H_2_O and CO_2_, which allows for an easy separation of the former by condensation.^530^ In the case of precombustion, swing adsorption processes are state of the art (see Section 4.1), having partly displaced the previously used chemical absorption-based technology.^365,531,532^ Direct air capture (DAC) is still under development, due to the difficulty of separating CO_2_ from an ultradilute source.^5,472,533,534^ In fact, techniques involving high interaction energies (formation of chemical bonds), such as chemical absorption and adsorption, are needed to capture CO_2_ from air and subsequently, a large energy input is needed for its recovery, which renders DAC a controversial approach.^472,535^ However, this technology is currently experiencing a rapid growth^6^ and close to 20 small scale facilities are already operational, with the first large-scale DAC facility being planned for 2024.^536^ As CO_2_ removal from precombustion streams and natural gas has already been discussed in previous sections (Sections 4.1, 4.6.1), the last paragraphs of this section will briefly deal with DAC and finally focus on the removal of CO_2_ from postcombustion streams.

DAC on zeolites has been studied and most of the works point at commercial and, in general, aluminosilicate zeolites not being promising adsorbents for this application due to water adsorption.^534,537−539^ In fact, a paper by Stuckert et al. showed that Li-LSX, K-LSX and 13X experience a 99, 88 and 100% drop in CO_2_ capacity at 80% relative humidity, respectively.^537^ Despite that, zeolites have been the adsorbent of choice for a recent start-up.^540^ A recent publication by Fu et al. shows interesting preliminary results on a Zn-exchanged chabazite, for which regrettably no cyclic experiments under humid conditions are shown.^541^

Postcombustion carbon capture deals with the separation of CO_2_ from flue gases containing 3–30% of CO_2_, the rest being N_2_, residual O_2_ and water, and a total pressure close to atmospheric pressure.^368,472^ Apart from the already mature aqueous amine absorption technology for CCS, promising technologies for CCS from postcombustion streams include chemical looping, adsorption, and membrane processes, but these need to be improved in order to allow for wider deployment of CCS processes.^472,527,528,542^ Carbon capture by adsorption may be carried out by pressure-vacuum swing adsorption or temperature swing adsorption techniques.^527,543^ In either case, the interplay betweeen adsorbent properties and process features determines the overall performance^331,544^ of a certain adsorbent–process pair. Furthermore, detailed energetic and cost analysis allows us to discern between potentially and practically applicable adsorbents.

The separation of CO_2_ from N_2_ has been studied on different adsorbents, including activated and microporous carbons, graphene-based materials, MOFs, amine-functionalized adsorbents, metal oxides and carbonates, zeolites, AlPOs, and SAPOs,^331−333,543,545−550^ but to the best of our knowledge, none of these adsorbents has been applied to a CO_2_ capture swing adsorption process that is competitive with current amine-scrubbing state-of-the-art techniques.

In what refers to zeolites and zeolite-type adsorbents (AlPOs, SAPOs, and titanosilicates), most research has focused on commercial type A, X, and Y zeolites, out of which zeolite 13X (NaX) is considered a benchmark adsorbent for postcombustion CO_2_ removal.^331,461,545−547,551,552^ Taking into account the conditions of a typical postcombustion stream (*P*_CO_2__ ≈ 0.15 bar, *P*_tot_ ≈ 1 bar, 25 °C ≥ *T* ≤ 100 °C^368,472,473^), it is not surprising that 13X is relatively adequate for this application. High adsorption capacity is needed at pressures close to ambient, along with high CO_2_/N_2_ selectivities. Furthermore, compared with chemisorbents, the energy required for its regeneration will be low. Nevertheless, its hydrophilicity is a great disadvantage that needs to be overcome with process modifications,^461,527^ such as a drying step prior to the CO_2_/N_2_ separation.^553^

Other materials with lower polarity, such as high- and pure-silica zeolites, AlPOs, and SAPOs, minimize water adsorption and the energy required for regeneration, while keeping promising selectivities and working capacities in some cases.^275,276,333,502,507,547,554−559^ A large effort is being put into finding optimal adsorbents, and optimal process configurations via computational screening of existing and theoretical adsorbents for the capture of carbon dioxide from flue gases.^331−333,558,560^ However, one frequent drawback of noncommercial adsorbents is the difficulty in scaling up their production from both technical and economical perspectives.^331^

A list of zeolitic adsorbents and relevant parameters for the separation of CO_2_ from N_2_ is provided in Table 4. Materials, such as SAPO-RHO (RHO),^507^ NaTEA-PST-20,^275^ Li-SSZ-13–6, Na-SSZ-13–6 (CHA),^555^ or SAPO-56 (AFX)^507^ present promising values of selectivities (>10) and working capacities (ca. 0.5 mmol/g) at relevant conditions, comparable with those of traditional 5A^521^ and 13X^522^ zeolites. Some AlPO materials with GIS and SIV structure are also predicted to be promising candidates.^333^

**Table 4:** Zeolitic Adsorbents for the CO_2_/N_2_ Separation^a^

Material	Structure	Si/Al ratio	Estimated framework negative charge^b^	*T* (°C)	*Q*_0.15 bar_ (mmol/g)	*Q*_1bar_ (mmol/g)	WC_0.15–0.07_ (mmol/g)^c^	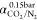	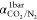	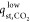 (kJ/mol)	ref·
5A	LTA	1	0.5	25	3.80	4.67	0.58	31	8.6	-	(521)
NaKA	LTA	1	0.5	25	2.38	3.38	0.47	172^d^	-	-	(561)
SAPO-RHO	RHO	-	<0.11	0	1.79	3.61	0.60	57	26^d^	32.5	(507)
SAPO-RHO	RHO	-	<0.11	35	0.73	2.13	0.33	-	-	32.5	(507)
Na-SAPO-RHO	RHO	-	<0.18	25	2.42	3.45	0.44	196^d^	37	42.5	(263)
Na-SAPO-RHO	RHO	-	<0.18	25	-	0.98^f^	-	107	-	42.5	(263)
PST-29	PWN	4.5	0.18	25	2.82	4.26	0.44	31	7.3	-	(502)
NaTEA-ECR-18	PAU	3.5	0.22	25	2.26	2.99	0.25	35	9^d^	53.0	(275)
NaTEA-ZSM-25	MWF	3.4	0.23	25	2.97	3.50	0.27	27	10^d^	65.0	(275)
NaTEA-PST-20	-	3.1	0.24	25	2.11	3.17	0.52	33	10^d^	44.0	(275)
CsTEA-ZSM-25	MWF	3.4	0.23	25	1.41	2.14	0.34	47^d^	9	27.6	(276)
CsTEA-PST-20	-	3.1	0.24	25	1.50	2.23	0.31	47^d^	11	35.0	(276)
K-MER-2.3	MER	2.3	0.30	25	2.21	2.99	0.26	95^d^	13	30	(264)
13X	FAU	1.2	0.45	25	3.01	4.69	0.62	67	18.4	37.2	(522)
Si-FER	FER	-	0	30	0.54	1.61	0.25	16.5	9.0	27.2	(554)
Si-MFI	MFI	-	0	30	0.38	1.66	0.19	13.3	10.7	24.2	(554)
Si-STT	STT	-	0	30	0.42	2.02	0.21	17.7	16.0	23.6	(554)
Si-CHA	CHA	-	0	30	0.39	1.92	0.20	15.4	12.3	23	(554)
Li-SSZ-13–6	CHA	6	0.14	30	3.52	5.09	0.64	29.1	9.5	44.2	(555)
Na-SSZ-13–6	CHA	6	0.14	30	3.54	4.95	0.63	33.5	9.9	43.0	(555)
r1.2KCHA	CHA	1.2	0.45	0	1.75	2.11	0.29	>100	80^d^	-	(258)
r1.9KCHA	CHA	1.9	0.34	0	0.62	0.81	0.14	>100	85	-	(262)
r1.9KCHA	CHA	1.9	0.34	30	1.13	1.49	0.20	>100	>100	-	(262)
AlPO-18	AEI	-	0	0	0.53	2.11	0.27	20.0	13.8	-	(556)
AlPO-18	AEI	-	0	20	0.27	1.36	0.14	14.1	13.1	-	(556)
AlPO-18^e^	AEI	-	0	25	0.34	1.86	0.17	15.7	23.6	26.8	(333)
SAPO-56	AFX	-	<0.15	0	2.77	5.46	0.86	33	10.5^d^	36.0	(507)
SAPO-56	AFX	-	<0.15	35	1.06	2.81	0.42	-	-	36.0	(507)
AlPO-53	AEN	-	0	0	0.86	1.92	0.37	55	25	-	(556)
AlPO-53	AEN	-	0	20	0.41	1.38	0.20	81	40	-	(556)
AlPO-53^e^	AEN	-	0	25	0.33	1.28	0.15	80	84	33.9	(333)
AlPO-GIS^e^	GIS	-	0	25	1.94	3.65	0.89	132	163	36.0	(333)
AlPO-ATT^e^	ATT	-	0	25	1.28	2.45	0.49	76	84	34.0	(333)
AlPO-SIV^e^	SIV	-	0	25	1.24	2.90	0.55	75	74	33.9	(333)

a Adsorption isotherm values have been extracted from the references where they were not explicitly displayed. Selectivities have been calculated therefrom, unless otherwise specified.

b The estimated framework negative charge gives analogous information as the Si/Al ratio and is defined in ref (152). Materials with values above 0.33 are considered to be highly polar.

c The working capacities have been calculated at CO_2_ pressures between 0.15 and 0.07 bar, i.e. VSA conditions.

d These selectivities have been directly taken from their respective references.

e Simulation results data of CO_2_/N_2_ 15:85 mixture.^333^

f Breakthrough data of a CO_2_/N_2_ 15:85 mixture, total pressure 1 bar.^263^

### 4.7 Removal of Miscellaneous Pollutants

Together with CO_2_, there are other gaseous compounds present in industrial streams that can act as pollutants of the environment, such as CO, NH_3_, NO_2_, NO, H_2_S, and SO_2_.^340,562^ Most of these are byproducts of industrial processes, especially combustion and chemical production, but some may as well be important starting materials (CO, NH_3_). In any case, their emission to the atmosphere needs to be minimized.

Carbon monoxide is a highly toxic compound that is produced along with hydrogen in steam-reforming processes and is mainly used as a raw material for chemical and metallurgic production. The incomplete combustion of fuels also leads to the formation of CO. Removal/recovery of CO is part of the process of hydrogen production, as presented in Section 4.1, and it may involve the use of H_2_-permeable membranes or adsorbents selective toward the other components of the mixture, e.g., zeolite 5A. Further separation of these components may be done by condensation/stripping/distillation steps.^562^ The separation of H_2_ and CO has been studied, as well, on zeolitic MFI-type membranes.^563,564^ Removal of CO from other mixtures, such as air or nitrogen, for its use as an inert may be done by Ag^+^-exchanged zeolites.^156,250^

Ammonia is a highly toxic and reactive gas that is produced at a very large scale and is used as a starting material in the production of fertilizers and chemicals. State-of-the-art techniques to prevent ammonia emissions to the atmosphere include water scrubbing and biological filtration;^565,566^ additionally, other techniques, such as membranes and adsorption are being studied.^567−577^ Zeolites have been known to adsorb ammonia since 1896, when Friedel published a work on the reversible adsorption of vapors in these materials.^27^ Since then, some studies on the adsorption of ammonia by zeolites have been issued.^28,572,573,578−580^ However, the large heat of adsorption of ammonia on traditional aluminosilicate zeolites is a drawback to their application,^581^ as it renders the hypothetical process not competitive with state-of-the-art techniques. Hence, it is not surprising that most attention has been put on MOFs regarding this separation in the last years.^574−577^ Nonetheless, several works study highly efficient zeolitic membranes of high silica MFI,^569,570^ and it has been recently evidenced that pure silica LTA and FAU zeolites are promising adsorbents for carrying out the removal of ammonia through a swing adsorption method.^571^

Hydrogen sulfide is a very toxic and corrosive gas that appears as a contaminant in fossil fuels (natural gas, petroleum, coalbed gas) and is also produced in the decomposition of organic matter (biogas).^338,582^ Its separation from these mixtures is essential for further use thereof, both as fuels and/or as starting materials, as it may cause corrosion and plugging of the equipment. Moreover, sulfur in its reduced state acts as a poison to catalysts containing noble metals, which may be used in refining and petrochemical processes (see Section 2.4.2). Therefore, desulfurization processes of fossil fuels have been developed to remove hydrogen sulfide and organosulfur compounds from the different refinery streams. The removal of H_2_S from gas streams is mostly done by amine scrubbing, especially for its joint removal with CO_2_.^562^ Adsorption on zeolites is preferred when the stream to be desulfurized contains a sufficiently low amount of carbon dioxide.^583^ Zeolites 4A and 5A were proposed for the removal of H_2_S, but they presented problems due to competitive CO_2_ and H_2_O adsorption and high energy demanding regeneration.^450^ Type X and Y zeolites have been evaluated, as well, for H_2_S and organosulfur compound removal,^584−586^ and so have ZSM-5 and natural clinoptilolites.^480,582^ However, the main problem with all these materials is that, apart from readily adsorbing H_2_S, CO_2_, and H_2_O, they also favor the formation of carbonyl sulfide (COS) at usual bed regeneration conditions (TSA) according to the reaction:^450,497,583^
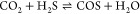
The formed COS still needs to be removed from the final product. Hydrophobic materials such as high-silica and pure-silica zeolites have been studied in both simulation and experiments, and they seem promising in this sense, as the equilibrium will not be shifted by preferential adsorption of water. Furthermore, the heat of adsorption of H_2_S on these materials is lower in comparison with traditional zeolites, and H_2_S/CH_4_ selectivities are still high, thus making regeneration easier without a loss in efficiency. Promising candidates from these studies are pure silica CHA and MFI zeolites.^587−589^ Titanosilicate ETS-2 has been proposed as a highly selective H_2_S sorbent, as well.^590,591^

Nitrogen oxides (mostly NO and NO_2_, frequently referred to as NO_*x*_) and sulfur oxide (SO_2_) are undesired byproducts of combustion processes and especially of coal and transportation fuels.^592−594^ They are toxic compounds that account for serious environmental problems, like smog and acidic rain, and also negatively affect human health. Their emissions are mainly prevented by proper treatment and selection of the fuel and by correctly setting the temperature and conditions of the combustion process. For instance, the development of the three-way catalytic converter for automotives has contributed to a decrease of the NO_*x*_ emissions through the method known as selective catalytic reduction (SCR),^594,595^ and the shift from coal toward other fuels for energy production has helped reduce SO_2_ emissions. Further control of SO_2_ emissions from coal burners can be done by a variety of well-implemented methods, which may be classified as regenerable and nonregenerable and usually involve the revalorization of the captured sulfur.^594,596^ Simultaneous catalytic removal and transformation of SO_2_ and NO_*x*_ has been also been studied and applied.^597,598^ There is, however, still an ongoing interest in developing adsorption processes with improved energetics for the removal of these compounds.

The removal and recovery of NO_*x*_ from nitric acid production waste streams was studied on 13X and mordenite in the late 1960s and early 1970s.^599,600^ Zhang et al. studied adsorption of NO on copper- and silver-exchanged ZSM-5 and mordenites and found that partially irreversible adsorption takes place.^601,602^ This phenomenon was later studied on transition-metal-exchanged zeolite A by Wheatley et al. for its use in medical applications as NO releasers.^603^ Zeolites NaY^604,605^ and Na-ZSM-5^606^ have been studied for NO_*x*_ removal from flue gas using PSA. Pd/SAPO-34 and Ca-β have been studied as low-temperature adsorbents of NO_*x*_ for their use prior to the SCR process in automotives.^607,608^

Being mostly based on chemical sorption, the current methods for SO_2_ capture would benefit greatly from a decrease in the energy required for regeneration of the sorbent. Thus, it is not surprising that a great research effort has been put into the search for SO_2_-selective adsorbents, which represent an alternative to the ones available.^594,609^ Zeolites are one type of adsorbent that has been studied for SO_2_ removal, and as in other cases, silicalite-1 and zeolites X and A are the ones that have received the most attention. In 1974 Breck published data on SO_2_ adsorption by relevant zeolites at the moment, such as KL, NaA (4A), CaA (5A), and NaX (13X), thus showing that zeolites could be potential SO_2_ adsorbents.^54^ Adsorption of SO_2_ was studied on hydrophobic zeolites DAY (dealuminated Y), silicalite-1, HZSM-5, and mordenite,^610−612^ and the conclusions of these works point at silicalite-1 being a suitable adsorbent for SO_2_ removal from flue gases. Kopaç et al. studied the adsorption of SO_2_ on commercial zeolites by means of pulse chromatography at temperatures above 250 °C and found that the affinity toward sulfur dioxide decreased in the order 4A > 13X > 5A.^613,614^ Gupta et al. found zeolite 5A promising for trace removal of sulfur dioxide at 70 °C.^615^ Srinivasan et al. studied a set of zeolites synthesized from fly ash and found that the largest SO_2_ adsorption capacity at room temperature was given by a mixture of sodalite and analcime.^616^ A computational screening of zeolitic structures carried out by Matito-Martos et al. suggested that zeolites with JRY, NAT, AFY, FAU, and SBE structures could be potentially applicable at certain conditions.^339^ Systematic studies dealing with SO_2_, NO, CO_2_, and N_2_ mixtures were carried out by Deng and co-workers, showing that zeolite 5A performed better than zeolite 13X in adsorbing the pollutants, especially SO_2_.^617,618^

### 4.8 Separation of Olefins from Paraffins

Light olefins (ethene, propene, butenes) are important raw materials for the production of polymers (e.g., polyethylene, polypropylene) and chemicals (e.g., ethylbenzene, cumene).^619^ They are produced mainly in catalytic cracking, steam cracking, thermal cracking, MTO, and catalytic dehydrogenation processes,^206,208,234,237,620−622^ along with other hydrocarbons. The separation of light olefins from the other products is performed by cryogenic distillation. Due to their close boiling points (see Table 2), the separation of these olefins, from their analogous paraffins (ethane, propane, butanes), is one of the most energy-consuming processes in the chemical industry.^1,623^ Therefore, finding alternative and complementary less energy-intensive methods for separating light olefins is of high interest.

Membrane-, absorption-, and adsorption-based technologies are promising candidates to replace the distillative separation of olefins from their analogous paraffins.^624^ These ways of separating olefins from paraffins may rely on differences in the physical and/or chemical properties of said molecules (see Table 2). Olefins present slightly smaller kinetic diameters and larger dipolar or quadrupolar moments and can establish chemical bonds (π-interactions) with some metallic species.^623^ In the case of adsorptive separation, this allows for different separation mechanisms, i.e., thermodynamic, kinetic, and molecular sieving, depending on the properties of the chosen adsorbent. Studies on the adsorptive separation of C2–C4 alkenes from alkanes have been carried out on different adsorbents, out of which zeolites and MOFs are the most promising.^363,624^ First, a joint discussion on the C2 and C3 fractions will be presented, and later, the more complex C4 fraction is discussed separately. The progress on MOFs is presented, as well, in order to give the reader a more detailed context on this separation.

Adsorbents that contain Ag and Cu, such as supported silicas and aluminas, exchanged zeolites (AgY), and several MOFs, may be thermodynamically selective toward the olefin thanks to a π-complexation mechanism.^155,625−627^ AgA is a material, which completely excludes ethane but adsorbs 2.3 mmol/g of ethene at 1 bar.^628^ Its working capacity is, however, extremely low, due to the almost square type I isotherm. Cu(I)–NaX is another material presenting a high ethene/ethane selectivity (ca. 5) and a low working capacity between 0.5 and 1 bar (ca. 0.3 mmol/g).^629^ The high heat of adsorption in these cases not only leads to low VSA/PSA working capacities but also makes regeneration more energy-demanding. Furthermore, the strongly adsorbed olefin can oligomerize inside of such adsorbents, giving rise to pore obstruction.

Thermodynamic selectivity may, as well, stem from physical interactions with the adsorbent. Adsorbents with a polar surface are selective toward the olefin, which is the most frequent situation.^623^ Aluminosilicate zeolites with FAU and LTA structures, more specifically, 13X,^630,631^ 4A,^632−634^ LiNaA,^635^ and 5A,^636,637^ along with some exchanged titanosilicates of types ETS-10^638^ and ETS-4^639^ have achieved high to very high selectivities (5–70) toward ethene and propene, although their working capacities in the range of 0.5–1 bar are low (<0.3 mmol/g). Zeolite 4A additionally presents a marked propene/propane kinetic selectivity.^633^ MOFs such as M-MOF-74 (M = Mg, Co, Ni),^630,640−642^ M_2_(m-dobdc) (M = Co, Fe, Ni or Mn; m-dobdc = 2,5-dioxido-1,4-benzenedicarboxylate),^643^ and NOTT-300^644^ are promising for ethene-selective separations,^624^ in which they present high to very high thermodynamic selectivities toward the olefins  and high to very high working capacities (0.5 mmol/g  2 mmol/g). (Cr)-MIL-101-SO_3_Ag presents large selectivities and working capacities for both C2 (,  = 0.6 mmol/g) and C3 (,  = 1 mmol/g) olefin separations.^645^ MOF MIL-101-Cr-SO_3_Ag presents a high selectivity (ca. 10) toward ethene and a notably high working capacity (0.9 mmol/g).^646^ However, the high production cost and reduced long-term stability of most of these materials hinder their deployment at a larger scale.

Several nonpolar MOFs have been reported to be thermodynamically selective toward ethane over ethene, although the selectivities are relatively low so far (<5) in comparison to ethene selective adsorbents.^624^ It is of high interest to achieve high working capacities and selectivities using paraffin-selective adsorbents, as these represent the lesser amount of the steam cracker product stream, and thus, their separation would require smaller adsorbent inventory and enable the direct production of a highly pure olefin stream.^624,647−650^ At the same time, by selectively adsorbing the alkane, the risk of olefin oligomerization is avoided.

Whereas thermodynamically selective adsorbents have received the most attention, it is kinetically selective and molecular sieving adsorbents that present the largest selectivities.^624,651^ Various pure- and high-silica zeolites and aluminophosphate-based materials presenting 8-rings, i.e., ITQ-29 (LTA), DD3R (DDR), ITQ-12 (ITW), Si-CHA (CHA), ITQ-3 (ITE), ITQ-32 (IHW), AlPO-34 (CHA), and Na-SAPO-17 (ERI), present extraordinarily high (700 – 46 000) propene/propane kinetic selectivities.^74,652−658^ Similarly, zeolite ITQ-55, a small-pore zeolite with extremely narrow pores, presents a very high ethene/ethane kinetic selectivity,^272^ which derives from an ethene adsorption-driven change in the framework structure. Further advantages of these pure-silica materials is that no reactions of the olefins will take place inside their nonpolar surface, and their hydrophobicity will prevent competitive adsorption of water and other relatively polar molecules. MOF [Ca(C_4_O_4_)(H_2_O)] presents an unprecedented molecular sieving effect, in which it adsorbs selectively only ethene and completely excludes ethane.^659^ Other MOFs, such as ZIF-8,^660^ ZIF-67 (ZIF = zeolitic imidazolate framework),^661^ Zn(ox)_0.5_(trz), and Zn(ox)_0.5_(atrz) (ox = oxalate, trz = 1,2,4-triazole, atrz = 3-amino-1,2,4-triazole),^662^ present kinetic selectivities of the order of 10^2^–10^3^ of propene over propane. These are probably not as high as for zeolites due to the higher flexibility of MOFs in general.

The separation of the olefins of the C4 fraction is more complex, as there are several different isomers to be taken into account. The C4 fraction includes butane, isobutane (2-methylpropane), 1-butene, *cis*-2-butene, *trans*-2-butene, isobutene (2-methylpropene), and 1,3-butadiene. They are coproduced along with ethene and propene in the steam cracking of naphta and as a byproduct in FCC.^622,663,664^ The increasing demand of the olefins of this fraction, especially isobutene (45%) and butadiene (40%), has led to targeting their production via other processes,^364^ such as MTO, low and high severity steam cracking,^664^ catalytic dehydrogenation of butane, or oxidative dehydrogenation of butene.^665^ Said processes yield products that consist of mixtures of C4 olefins and paraffins in various proportions.^364,665^ Therefore, the separation of the C4 fraction is necessary for the further use of several components. This separation cannot be carried out via simple distillation due to the close boiling points of the different species (see Table 2). Extractive distillation is used instead using a specific mass separating agent for each step.^7^

Butadiene polimerizes readily under a wide set of conditions, and thus it must be handled and kept in the absence of oxygen and acids. It is separated first via extractive distillation or liquid–liquid extraction. Then, further extractive distillation may be used to separate butanes from butenes. Isobutene is separated by reactive distillation in the forms of *tert*-butanol or methyl *tert*-butylether. The remaining linear butenes and butane are separated by either extractive distillation or molecular sieving (the reference containing information regarding said molecular sieve could not be accessed by the authors of this review).^664^

Adsorptive separation of the components of the C4 fraction has been studied on different materials, such as zeolites, mesoporous silicas and MOFs.^363,364^ The C4 olefins maintain the typical olefin interactions with metals (such as π- and/or electrostatic interactions) and also general trends, such as larger diffusivity thereof compared to the analogous paraffins if the pore size is adequate. Nevertheless, new levels of hierarchy arise, and each and every one of the C4 isomers behaves differently upon adsorption on microporous frameworks.

The separation of butadiene from the other C4 components using zeolite 13X was patented in 1976,^666^ but this process is not used currently, as it does not match the present purity requirements of butadiene.^363^ An adsorption process for the separation of butenes from butanes on 13X using a C5 or C6 paraffin as a desorbent has been patented by Kim et al. recently.^667,668^ The separation of the other C4 components under thermodynamic control has been studied on commercially available FAU (12-rings) and MFI (10-rings) zeolites, such as 13X,^669^ NaY,^670^ ZSM-5, and silicalite-1.^671^ The adsorption on 13X follows the order *cis*-2-butene > 1-butene > *trans*-2-butene > butane.^669^ On NaY, the same order is kept (isobutene > *cis*-2-butene > 1-butene > *trans*-2-butene > butane),^670^ which indicates that enhanced interactions are established by isobutene, followed by *cis*-2-butene. MFI-structured materials, such as ZSM-5 and silicalite-1, present a limited diffusivity of the branched compounds in comparison to the linear compounds.^672,673^ Furthermore, MFI materials adsorb larger quantities of 1-butene than *n*-butane.^671^

Interactions with transition and noble metal cations, such as Ag and Cu, have been exploited to achieve high selectivities toward butadiene. Zeolite AgY presents a large selectivity of 1-butene and butadiene over butane.^674,675^ Further selectivity was observed for NaY zeolites of Si/Al ratios between 6 and 15, in which butadiene was adsorbed at higher amounts.^675^ Zeolite Cu(I)Y was studied by the same authors,^676^ and better butadiene/1-butane selectivities than on AgY were found, with the additional advantage of Cu(I) not being sensitive to the presence of contaminants such as H_2_ or H_2_S.

However, similarly to the C2 and C3 fractions, it is kinetically selective low polarity small-pore zeolitic adsorbents that appear to be more promising.^363,364^ In this case, the preferred linear compounds tend to be 1,3-butadiene and *trans*-2-butene, and the least adsorbed one is butane. Isobutane and isobutene are usually excluded from small-pore zeolites. Pure- and high-silica zeolites with CHA, DDR,^677−679^ IHW,^657^ RRO,^680^ and AlPOs and SAPOs with ERI structure^681^ have been studied for this application. Si-CHA, AlPO-34, DD3R, and SAPO-17 are considered to be promising materials,^364,682^ and Si-CHA and AlPO-34 were patented for ExxonMobil in the 2000s.^677,678^

### 4.9 Separation of Linear, Branched, and Multibranched Paraffins

Gasoline is a liquid hydrocarbon mixture which consists mainly of hydrocarbons in the C4–C12 fractions and is one of the most widely used fuels. The octane number (ON) is a measure of the performance of the gasoline upon combustion in an internal combustion engine (ON of ca. 100 is desired), and it is regulated by official institutions. The ON of gasoline depends on its composition, in which some of its components, such as branched paraffins, aromatics, or olefins, increase the ON of the mixture.^683^ Nonetheless, some of these components, such as benzene, aromatics, and olefins, are restricted due to their environmental and/or health hazards.^684^ This leaves branched paraffins as the component of choice to meet ON specifications. Hydroisomerization of straight run naphtha (mostly linear C4–C10 paraffins) is an effective method of obtaining higher ON components for the gasoline blend. These are reacted with hydrogen in the presence of a highly active supported metal hydrogenation catalyst to yield the desired multibranched products. Due to equilibrium limitations, low temperatures are needed in order to minimize hydrocracking.^175,684,685^ A strategy which prevents hydrocracking from taking place and thus increases the yield and productivity of the unit includes separation of the branched products from the effluent and recycling of the linear and monobranched hydrocarbons to the head of the unit.^686,687^ The separation of linear from branched isomers is done by adsorption using zeolite 5A as the adsorbent. This zeolite has been implemented in liquid- (SMB) and vapor-phase (VSA/TSA) hydrocarbon separation processes since the 1960s.^253,254,427,688−690^ In fact, molecular sieving separations of linear and branched paraffins were one of the first major industrial successes of zeolites.^174,248,427,691^ Other zeolites, such as X, Y, and ZSM-5, have been commercialized for this purpose, as well.^692−695^

However, if applied at the exit of the hydroisomerization unit, the ideal target is the separation of linear and monobranched hydrocarbons from multibranched ones, as it would increase the efficiency of the whole process by recycling both low-octane linear and monobranched hydrocarbons to the head of the unit and yield a multibranched product-enriched raffinate with a high ON. Several materials have been studied and patented for this purpose, out of which silicalite-1 (Si-MFI) and other materials with MFI structure have been most frequently considered.^334,696−704^ Other zeolites with diverse structures, such as AFI,^705^ AEL, ATO, BEA, FAU, FER,^694,696^ ATS, CFI,^706,707^ EUO, MWW, NES,^13,702,708,709^ MEL, MRE, and MTT,^703^ have been patented for this separation as well, but none of them clearly surpasses Si-MFI.^13^ Recently, pure silica zeolites Si-STW^710^ and EMM-17^711^ and MOF Fe_2_(BDP)_3_ (BDP^2–^ = 1,4-benzenedipyrazolate),^712^ have been proposed as promising adsorbents for this separation. Zeolite Si-STW is superior to Si-MFI at ambient temperature in both adsorption capacity (ca. 30% increase) and kinetic selectivity toward the linear and monobranched compounds in the C5 fraction. Further selectivity between dibranched compounds was found, in which quaternary C-atom-containing isomers of the C5 and C6 fractions were completely excluded.^710^ EMM-17 can be used to separate C6 isomers at ambient temperature as determined from breakthrough experiments. The separation is kinetically controlled, with *n*-hexane and 2,2-dimethylbutane being the fastest- and slowest-diffusing isomers, respectively.^711^ Fe_2_(BDP)_3_ presented slightly better performance (0.54 mol/L) in terms of the 92 RON productivity than top-performing zeolites with MWW (<0.52 mol/L) and MFI (0.51 mol/L) structures.^712^

### 4.10 Separation of Benzene Derivatives

Catalytic reforming of naphtha (mainly C6–C10 *n-*paraffins) results in a product rich in benzene, toluene, xylenes (dimethylbenzenes), and ethylbenzene,^204^ frequently referred to as BTX. There are several possible isomers of xylene, i.e., methaxylene, orthoxylene, and paraxylene, also referred to as *m*-xylene, *o*-xylene, and *p*-xylene (see Figure 5). Out of these isomers, *p*-xylene is the most important commercially. It is a chemical feedstock in the production of the widely used polyester polyethylene terephthalate.^175^ Apart from catalytic reforming of naphtha, it may also be selectively produced in processes where the catalyst is shape selective (mostly ZSM-5, see Section 2.4.2), such as xylene isomerization, toluene disproportionation coupled with transalkylation, and alkylation of toluene with methanol. A high purity of *p*-xylene is desired, and thus a highly efficient separation step is needed in any case.

Benzene and toluene are easily separated by distillation. Xylenes and ethylbenzene (C_8_H_10_ isomers) may be further separated in a super fractionation unit, where orthoxylene is separated from the other three components.^14,206^ However, the very close boiling points of these components (see Table 2) make their separation by distillation highly energy demanding and, save for that of *o*-xylene, impractical. On the contrary, the melting points of the isomers are considerably different, allowing a separation of *p*-xylene using crystallization techniques at low temperatures.^14^ The crystallization temperature (from −90 to −53 °C) is lower than that of the pure components, due to the eutectic mixture that results.^713^ Another way of separating these compounds is via adsorption and, more specifically, via molecular sieving, taking advantage of their different sizes.^14,544^ Paraxylene is the smallest of the three isomers, and thus, it may be adsorbed on a material with the right pore size, while the other components of the mixture may be excluded.

The separation of *p*-xylene from *m*-xylene and ethylbenzene takes place in the liquid phase using simulated moving bed adsorption.^544,714^ Even though zeolites with MFI structure are frequently used in catalytic processes to selectively produce *p*-xylene, the identical kinetic diameters of *p*-xylene and ethylbenzene prevent MFI zeolites from being used as adsorbents for their separation. Additionally the adsorption capacity of MFI is low in comparison to other zeolites. FAU-structured materials are the adsorbents of choice for this separation, and most research has focused on them. Their selectivity can be tuned by appropriate ion exchange.^715^ Bicationic K- and Ba-exchanged zeolites X or Y are the preferred adsorbents in this case, as they provide sufficient equilibrium selectivity toward *p*-xylene and adequate mass transfer.^14^ Other adsorbents, such as MRE,^716^ MEL, and MWW zeolites^717^ and several MOFs^718^ have been proposed for the separation, but none of them is under industrial use.

### 4.11 Separation of Bioalcohols from Fermentation Processes

Environmental concern and the future shortage of petroleum-derived products have boosted the research and production of renewable fuels and chemicals. Short-chain alcohols, such as ethanol, 1-butanol (*n*-butanol), and more recently 2-methylpropane (isobutanol) can be produced from renewable resources via biological pathways and serve as biofuels and starting materials for the production of important chemicals.^3,434,719^ For instance, producing light olefins from bioalcohols by dehydration is a highly interesting approach,^720,721^ for which some processes have been already developed, such as Axen, Total, and IFPEN’s ATOL process.^722^

Bioethanol can be produced by fermentation from starch, sugars, or even cellulose (after hydrolysis). The resulting aqueous fermentate contains 8–14 vol % of ethanol, which needs to be separated for further use. Currently, its separation involves at least two steps, the first of which consists of distillation and leads to the formation of a distillate with a maximum ethanol content of 95.5 wt %, due to the ethanol–water azeotrope.^434,723^ The second step may involve a technique, such as low-pressure distillation, azeotropic distillation, extractive distillation, extraction, pervaporation, or adsorption, out of which pervaporation, extractive distillation using CaCl_2_, and adsorption are the less energy-intensive methods.^434^ Drying of this azeotropic mixture by adsorption may be carried out using zeolite 3A.^251,432,433^

Biobutanol, chemically speaking, 1-butanol, is an excellent biofuel with analogous properties to gasoline, and it serves as a platform molecule for the production of important chemicals, such as butenes. 1-Butanol can be produced from fermentation of starch and sugars, in what is known as the ABE (acetone, butanol, ethanol) fermentation, first patented by Chaim Weizmann (known under the name Charles Weizmann in Britain, where he carried out his research activities) in the 1910s.^724−727^ This process has been intermittently used throughout the years to obtain 1-butanol and/or acetone and is currently of great practical interest.^3,728,729^ Different strains of bacteria of the class clostridia, e.g., *Clostridium acetobutylicum* and *clostridium beijerinckii*, can perform this fermentation. The process is carried out in anaerobic conditions, and the product consists of a diluted aqueous solution (< 3wt %) of acetone, butanol, and ethanol in a 3:6:1 molar ratio, respectively.^730^ Along with the liquid products, some CO_2_ and H_2_ are produced in the fermentation.

The recovery of 1-butanol from the fermentation broth was originally carried out by distillation. However, mainly due to its low concentration in the product, this turns out to be a highly energy-intensive method and requires a high-energy integration and capital cost.^729,731^ Alternatively, it can be carried out following different methods, such as extraction, gas stripping, pervaporation, or adsorption, out of which the last two seem the most promising.^731,732^ Many studies on liquid-phase adsorptive separation of butanol from the ABE product have been carried out using a variety of adsorbents,^730^ such as activated carbons,^341,733−737^ polymeric resins,^734,736,738−741^ zeolites,^341,736,737,740,742−747^ or MOFs.^735,737^ In 2014 Abdehagh et al. proposed the combination of gas stripping and adsorption (i.e., vapor-phase adsorption) as an effective recovery method.^732^

The isolation of 1-butanol from the fermentation broth using vapor-phase adsorption on microporous materials has since been studied by different groups on activated carbons,^748−750^ zeolites,^748,751−754^ and MOFs.^754−756^ The effect of CO_2_ as a carrier gas has been considered in some of these works.^754,756^ As can be seen, zeolites have been used as adsorbents for liquid- and vapor-phase separations, with silicalite-1 being the most frequently studied material, probably due to it being the first pure silica zeolite available.^757,758^ Cavity-like zeolites, such as pure silica LTA (Si-LTA) and SAPO-34, i.e., CHA-structured SAPO, were used in combination by van der Perre et al. to achieve an unprecedentedly high recovery and purity of 1-butanol.^751^

Renewable isobutanol (bioisobutanol) can be produced via fermentative and nonfermentative methods, which have been developed only recently.^719,759−761^ Isobutanol is a very important platform molecule that can be used as a starting material in the production of olefins (butenes) and aromatics first and polymers and gasoline additives (MTBE) finally.^762^ The recovery of bioisobutanol from the aqueous phase is currently done by gas stripping and solvent extraction^763,764^ or by flash evaporation, followed by condensation and distillation.^759,765^ but alternative methods, such as extractive distillation using K_2_CO_3_^766^ and adsorption, have been proposed. In the case of adsorption, a pure silica Beta zeolite has been used for the vapor-phase recovery of isobutanol from its mixtures with ethanol and water, achieving high selectivities and promising separation performance.^767^

## 5 Summary and Future Prospects

Zeolites are microporous crystalline materials widely used as catalysts, adsorbents, and ion exchangers. The main characteristics, preparation methods, and applications have been presented. The basic concepts regarding adsorption and separation processes on zeolites have been summarized. The relevance of zeolites as adsorbents has been examined, and the most important applications of zeolites in adsorption and separation science have been reviewed. Zeolites and zeolite-like materials are state-of-the-art adsorbents for applications, such as the purification of hydrogen from SMR (zeolite 5A), the drying of industrial streams (zeolites 3A and 4A), the small- to intermediate-scale air separation (LiLSX), the separation of nitrogen from methane (ETS-4, CTS-1), the separation of linear hydrocarbons from isomer mixtures (5A), and the separation of *p*-xylene (K-, Ba- X and Y zeolites).

Overall, a trend can be seen where well-established zeolitic adsorbents are kept for ongoing industrial processes, and new zeolitic materials are studied for emerging adsorptive separations, such as the separation of hydrogen isotopes, carbon dioxide capture, separation of olefins, removal of pollutants, and separation of bioalcohols. In the case of the well-established zeolitic adsorbents, most of the research is directed toward process improvement. Process intensification should be considered for adsorptive separations using zeolites, e.g., by studying tandem processes, where adsorption and reaction are coupled. Two major advantages can be obtained from such approaches, the first being an equilibrium displacement of the reaction (sorption-enhanced reaction) and the second better heat integration of the whole process. Adsorbent conformation to improve process dynamics and mass transfer is another research topic which deserves careful consideration.

On the other hand, the emerging applications each present specific research questions and needs:

- The separation of N_2_/CH_4_ is carried out by ETS-4 materials at a small scale, yet the challenge to replace cryogenic distillation at a large scale remains. Research should be directed toward finding materials with high enough selectivities and improved regenerability.
- The widely studied separations of CO_2_ from N_2_ or CH_4_ have been carried out using zeolites of different types. In the case of CO_2_/CH_4_, new studies should be directed toward low polarity small pore zeolites, which prevent water competition and improve process performance. In the case of CO_2_/N_2_, the use of medium Si/Al ratio small pore zeolites is encouraged. Molecular sieving and trapdoor phenomena should be exploited while keeping minimal competition with water and high regenerability.
- The olefin/paraffin separation is one of the most important industrial separations and one that is still carried out via distillation, which is a highly energy-demanding process, due to the close boiling points of the compounds to be separated. The highest kinetic separation factors of olefins over paraffins have been found using small pore pure-silica zeolites as adsorbents, which makes them very promising.
- Recent studies on hydrocarbon separations by pure-silica medium pore zeolites open up the possibility of separating linear and monobrached from dibranched hydrocarbons, including olefins.
- The combination of gas stripping and adsorption for the recovery of alcohols from fermentation broths has resulted in a very efficient approach. Pure silica zeolites pose as a promising alternative to the currently implemented methods, which are mostly based on distillation. Lowering the energy required for these separations opens the door to the truly sustainable use of bioalcohols as biofuels and chemical feedstocks. In this sense, the research focus should be put into testing a wider set of pure-silica adsorbents and on improving the regeneration procedure.
- The highly challenging separation of hydrogen isotopes has been tried by many, and the success ratio has been low to the moment. Nonetheless, it is important that new adsorbents and membranes continue to be evaluated for this application. Adsorbents with pores smaller than 3 Å are the most obvious choice for pursuing a quantum sieving-based separation.

As can be seen, pure silica zeolites have gained importance for hydrocarbon and bioalcohol separations, where undesired reactions need to be avoided, and the hydrophobicity prevents competition with water adsorption. This is also true for CO_2_ adsorption, where in addition small pore zeolites presenting gating effects are receiving the most attention. Small pore zeolites featuring extremely small pore openings (< 3 Å) are the most relevant for hydrogen isomer separation, CO_2_ capture, and ethylene/ethane separation.

Commercial availability has been and still is one of the primary reasons why certain adsorbents are tested for new applications. With the progressive addition of more zeolitic materials to the spectrum of commercial absorbents, it will be easier for adsorption researchers to acquire and test these. Nonetheless, the input from zeolite synthesis research groups is especially valuable, as the technology and know-how needed to produce new and existing zeolites that are not commercially available is not accessible to everybody. Thus, it is not surprising that joint research by zeolite and adsorption experts has resulted in some of the most promising discoveries in the field of zeolitic adsorbents.

The active search for new structures and new compositional variants of existing structures is mostly driven by previous observations on materials sharing similar properties. However, the trial-and-error approach is not always truly successful in producing a scientific breakthrough but rather gradual improvements or purely nonapplied knowledge. Therefore, the identification of promising structures for a certain separation can be greatly accelerated by computational screening, which despite the simplifications needed correctly predicts general trends. Additionally, these computational studies serve as a major driving force to achieve the synthesis of new materials. We also firmly believe that the enormous quantity of accumulated adsorption data comprises a solid starting point for data-mining approaches, which have proven successful in the field of zeolite synthesis.

As mentioned throughout the review, a major drawback of new adsorbents is their production and scaling-up cost. In some cases, the added value of the product of a separation accounts for the high cost of the sorbent, provided that the regenerability is sufficiently good. However, in most cases adsorbents that are very promising in the laboratory never make it to the next stage of practical development. These issues can be addressed by aiming toward robust, hydrophobic adsorbents which can be produced without the need of an expensive OSDA.
